# Chlamydial membrane vesicles deliver the beta barrel outer membrane protein OmpA to mitochondria to inhibit apoptosis

**DOI:** 10.1371/journal.ppat.1013247

**Published:** 2026-02-02

**Authors:** Andreea Mesesan, Henry Oehler, Collins Waguia Kontchou, Aladin Haimovici, Martin Helmstädter, Oliver Kretz, Oliver Schilling, Stefan Tholen, John Atanga, Irina Nazarenko, Ulf Matti, Jonas Ries, Ian E. Gentle, Georg Häcker

**Affiliations:** 1 Institute for Medical Microbiology and Hygiene, University Medical Center and Medical Faculty, University of Freiburg, Freiburg, Germany; 2 Spemann Graduate School of Biology and Medicine (SGBM), University of Freiburg, Freiburg, Germany; 3 Faculty of Biology, University of Freiburg, Freiburg, Germany; 4 EMcore, Renal Division, Department of Medicine, University Freiburg Medical Center, Faculty of Medicine, University of Freiburg, Freiburg, Germany; 5 III. Department of Medicine, University Medical Center Hamburg-Eppendorf, Hamburg, Germany; 6 Institute for Surgical Pathology, University Medical Center and Medical Faculty, University of Freiburg, Freiburg, Germany; 7 Proteomics Platform – Core Facility (ProtCF), Medical Center – University of Freiburg/ Medical Faculty – University of Freiburg, Freiburg, Germany; 8 Institute for Infection Prevention and Control, University Medical Center, Medical Faculty, and Biology Faculty University of Freiburg, Freiburg, Germany; 9 Cell Biology and Biophysics Unit, European Molecular Biology Laboratory (EMBL), Heidelberg, Germany; 10 Max Perutz Labs, Department of Structural and Computational Biology, University of Vienna, Vienna, Austria; 11 Signalling Research Centres BIOSS and CIBSS, University of Freiburg, Freiburg, Germany; Virginia Commonwealth University School of Medicine, UNITED STATES OF AMERICA

## Abstract

*Chlamydiae* are obligate intracellular bacteria that inhibit mitochondrial apoptosis to maintain integrity of the host cell. We have previously reported that a chlamydial outer membrane β-barrel protein, OmpA, can during ectopic expression inhibit mitochondrial apoptosis through direct interaction with the BCL-2-family effectors BAX and BAK. We here show that OmpA from *Chlamydia trachomatis* (*Ctr*) uses membrane vesicles for its delivery to the outer mitochondrial membrane during *Ctr* infection. Using a number of imaging and fractionation techniques, we show that OmpA during infection reaches mitochondria and is inserted into mitochondrial membranes. Chlamydia derived vesicles (CDV) from *Ctr*-infected cells contained OmpA as well as other outer membrane proteins and LPS. When added to uninfected cells, CDVs fused with mitochondrial membranes, causing the interaction of OmpA with BAK and the cytosolic retro-translocation of BAX. CDV addition to uninfected cells also protected the cells against apoptosis. We previously showed that OmpA works in co-ordination with VDAC2 to block apoptosis and here propose a structural model of this BAK inhibition by OmpA that reenacts the inhibition of BAK by VDAC2. The results provide evidence that OmpA from *Chlamydia*, as well as the structurally similar ortholog from the related *Simkania*, specifically exploits its relationship to mitochondrial porins to protect the infected cell against apoptosis and to enable intracellular growth of the bacteria in human cells.

## Author summary

In this study, we investigated how a protein from *Chlamydia* called OmpA—which interferes with apoptosis, a form of controlled cell death—can reach mitochondria. *Chlamydia* live inside human cells within a membrane-bound compartment called an inclusion. Apoptosis is activated at mitochondria, and we previously found that OmpA expressed in cells can travel to mitochondria and block this process. Here, we show that in infected cells *Chlamydia* use vesicles derived from their own membranes to transport OmpA across the inclusion and through the host cell to mitochondria. Mitochondria are thought to have evolved from ancient bacteria and contain unique membrane proteins called beta barrels, found only in their outer membrane and in certain bacteria. OmpA shares this structure, which allows it to access and interact with mitochondrial proteins. We propose that OmpA binds to BAK, a pro-apoptotic protein at mitochondria, in a similar way to the human beta-barrel protein VDAC2, which naturally restrains BAK activity and cell death. By mimicking this interaction, OmpA prevents the cell from initiating apoptosis, enabling *Chlamydia* to survive longer inside human cells. Our findings reveal a strategy by which bacterial pathogens exploit the evolutionary origins of mitochondria to manipulate cell death and promote their growth.

## Introduction

*Chlamydia trachomatis* (*Ctr*) is a bacterial pathogen with considerable medical importance. It is very common worldwide as an agent of sexually transmitted disease [1] and in some areas of the world relevant as the agent of ocular trachoma [2]. *Ctr* infects epithelial cells and, as an obligate intracellular bacterium, it depends on the integrity of the host cell. Apoptotic death of an infected cell has been noted many years ago as a defense mechanism against viral infection [3], and experimentally induced apoptosis disrupts the chlamydial developmental cycle as expected [4]. All tested species of the genus *Chlamydia* express an anti-apoptotic activity: cells infected with *Chlamydia* are profoundly protected against experimental pro-apoptotic stimuli, as first shown for infection with *Ctr* [5]. *Parachlamydia*, a *Chlamydia*-like organism that infects free-living amoebae, induces apoptosis and cannot grow in human or insect cells. When however the mitochondrial apoptosis pathway was disabled, growth of *Parachlamydia* in human cells was observed [6]; in insect cells, caspase inhibition had a similar effect [7]. Current knowledge therefore suggests that human cells can undergo apoptosis to defend the organism against the infection with *Chlamydia*, and human pathogenic chlamydial species had to evolve an anti-apoptotic strategy. Related *Chlamydia*-like species, whose hosts have no apoptosis system as in the case of *Parachlamydia* and amoeba, do not have the need of an anti-apoptotic mechanism and therefore cannot grow in human cells.

How the anti-apoptotic activity of *Chlamydia* works on a molecular level has been the focus of numerous studies, mostly using *Ctr*. *Ctr* induces many changes to cell signaling and metabolism, and a number of these changes have the potential to act in some anti-apoptotic way. However, the data point to a mechanism that operates at a central signaling step of the apoptotic pathway. The apoptosis signaling pathway is activated by numerous stimuli and converges on the activation of effector caspases, mostly caspase-3. Caspase-3 can be activated by caspase-9 in the mitochondrial pathway and by caspase-8 in the death receptor pathway [8]. Infection of human cells with *Ctr* blocks only mitochondrial apoptosis but not the death receptor pathway in the absence of a mitochondrial contribution [9]. In the mitochondrial pathway, the decisive step is mitochondrial outer membrane permeabilization (MOMP), which leads to the release of intermembrane space proteins (in particular cytochrome *c*) that in the cytosol induce the activation of caspase-9 and the downstream steps of apoptosis. MOMP is regulated by the BCL-2 family of proteins. Within this family, one anti-apoptotic group (BCL-2 and its homologous ‘BCL-2-like’ proteins) directly bind and inhibit the two pro-apoptotic groups of proteins, BH3-only proteins and effector proteins (BAX and BAK). Upon their activation, BAX/BAK are the most downstream proteins of the pathway to MOMP, as they directly form pores in the outer mitochondrial membrane [10,11].

Small molecule inhibitors of BCL-2-like proteins (so-called BH3-mimetics) have become available. At least in cell lines, they inhibit for instance BCL-2, which causes the direct activation of BAX/BAK, bypassing any upstream signals. This experimental approach has been used to map the anti-apoptotic activity of *Ctr*. As said above, there may be individual ways of apoptosis inhibition by *Ctr* infection in some more upstream pathways that also regulate apoptosis. The use of BH3-mimetics however has allowed us to identify a strong anti-apoptotic activity in *Ctr*-infected cells downstream of BCL-2-like proteins [12]. This activity involved direct interaction of BAK with a protein generated during *Ctr* infection, which blocked BAK-activation (measured by conformational changes and oligomerization) at identifiable steps. The apoptotic activity of BAX, on the other hand, is in part counter-regulated through driving its retro-localization from mitochondria to the cytosol, and this activity was enhanced during infection, removing BAX from mitochondria and preventing its pro-apoptotic activity [12].

Intriguingly, molecularly similar anti-apoptotic activity has previously been described for a mitochondrial porin, VDAC2. Porins are β-barrel proteins inserted in the outer mitochondrial membrane or in the outer membrane of Gram-negative bacteria, where they regulate solute flux across the membrane. *Ctr* has a highly expressed beta barrel protein, the major outer membrane porin (MOMP; we will here use the gene name OmpA to avoid confusion with mitochondrial outer membrane permeabilization). The small size of the predicted barrel of OmpA has led to some doubt as to its function as a porin however. We have in previous work expressed OmpA in (uninfected) HeLa cells and found that it localized to mitochondria where it inserted into the membrane. Furthermore, OmpA expression (in the absence of infection) precisely phenocopies the anti-apoptotic effect of *Ctr* infection on a molecular level and that this required to a certain extent the presence of VDAC2 as VDAC2 deficient cells were less protected from BH3 mimetic drugs [12]. This study suggested that OmpA may be a major anti-apoptotic effector of chlamydial infection and that it functions in coordination with VDAC2 in the mitochondrial outer membrane. This is an attractive model where *Chlamydia*, as a Gram-negative bacterium, would use its evolutionary relationship to mitochondria, to inhibit apoptosis by mimicking the anti-apoptotic activity of VDAC2.

The obvious difficulty with this model is the question how OmpA could translocate to mitochondria. *Ctr* replicates in the host cell within a cytoplasmic vacuole (the inclusion), so OmpA would have to be released from the bacteria, cross the inclusion membrane and traffic through the cell to reach mitochondria. However, immunostaining has identified OmpA outside the inclusion probably in vesicles, most likely outer membrane vesicles (OMVs) that appeared to move through the cell [13,14]. Even though mechanistically unclear, OMVs from various bacterial species have been shown to traffic through mammalian cells and sometimes to target mitochondria, as shown for OMVs from *E. coli* [15] and from *Neisseria gonorrhoeae* [16]. We therefore here tested the hypothesis that OmpA traffics to mitochondrial outer membranes during *Ctr* infection through vesicular trafficking where it acts as an anti-apoptotic protein in coordination with VDAC2.

## Results

### Inhibition of apoptosis by bacterial porins

**Fig 1:**
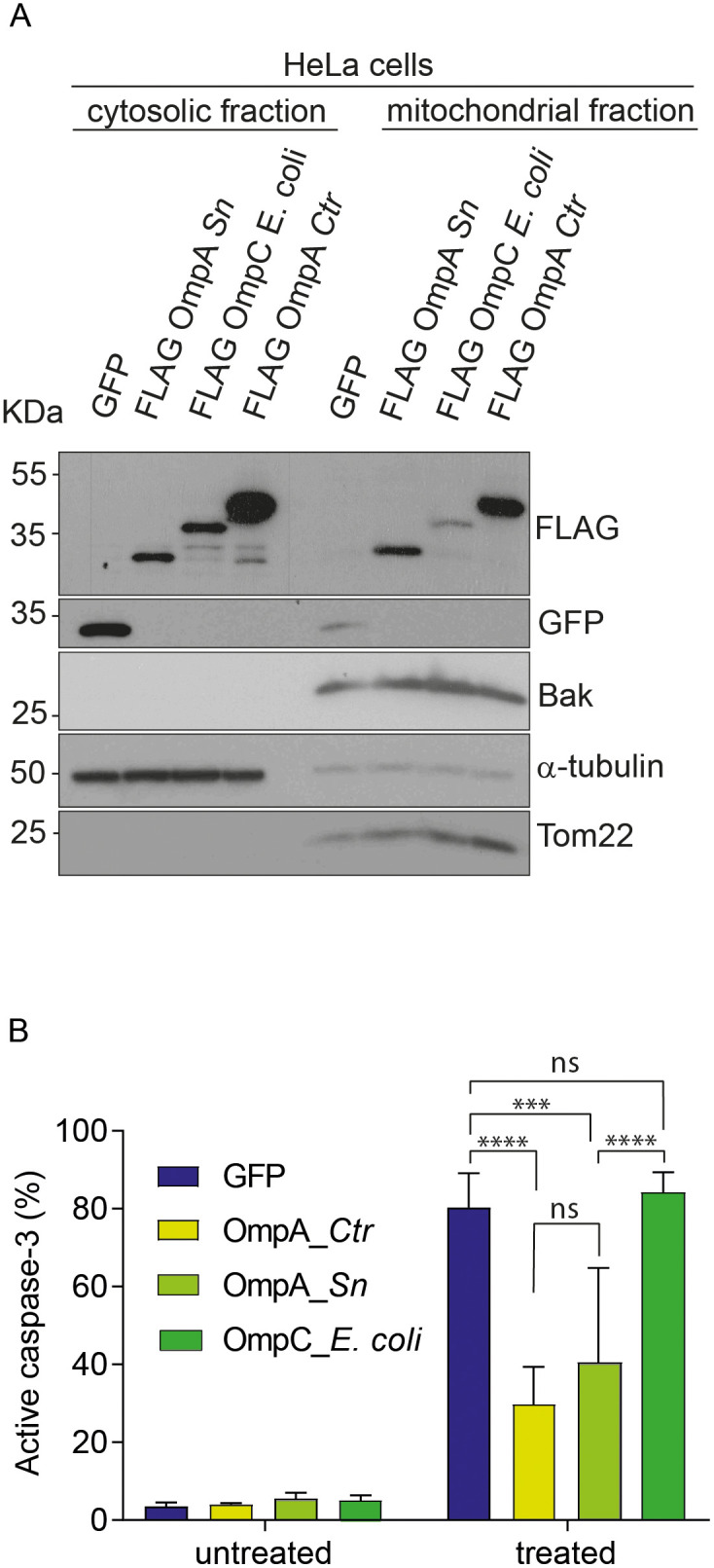
Mitochondrial localization of bacterialβ-barrel proteins. A, HeLa cells constitutively expressing GFP, FLAG-OmpA from *Simkania negevensis* (*Sn*), FLAG-OmpC from *E. coli*, or FLAG-OmpA from *Ctr* were fractionated, and mitochondrial and cytosolic fractions were subjected to anti-FLAG or anti-GFP Western blotting to analyze the subcellular localization of GFP, OmpC or OmpA. TOM20 and BAK were used as mitochondrial markers, α-tubulin as a marker of the cytosol. B, *Sn*OmpA, *E. coli* OmpC and *Ctr* OmpA were ectopically expressed in HeLa cells. Cells were treated with ABT-737 (1 μM) and S63845 (500 nM) for 4h. Cells were fixed, permeabilized and stained for active caspase-3 to measure the number of apoptotic cells. Data are representative of three independent experiments. Error bars represent SEM and significance was tested using 2-way ANOVA (***, p < 0.001, ****, p < 0.0001).

All tested *Chlamydia* species inhibit apoptosis. All of these have OmpA proteins that are closely related to *Ctr* OmpA. The only *Chlamydia*-like bacterium outside the *Chlamydiaceae* that is known to inhibit apoptosis is *Simkania negevensis* (*Sn*) (order *Simkaniales*) [17]. Intriguingly, OmpA genes in a non-*Chlamydiales* species in the data base are only found in *Sn* and in some not further characterized species of the *Simkaniales* and the *Rhabdochlamydia*. While lacking some regions of *Chlamydia* OmpA, *Sn*OmpA shows a high level of similarity to the OmpA from various *Chlamydia* species, particularly within the beta barrel strands (S1A Fig). We expressed *Sn*OmpA in human HeLa cells and tested for anti-apoptotic activity. As a control, we chose a random bacterial porin, OmpC from *E. coli* (*Ec*). As we reported before [12], *Ctr*OmpA localized to the mitochondrial fraction of the cells to a substantial degree (Fig 1A). *Sn*OmpA also showed strong mitochondrial localization while *Ec*OmpC mostly remained cytosolic (Fig 1A). Expression of either *Ctr*OmpA or *Sn*OmpA protected the cells against apoptosis induced by BH3-mimetics, i.e., downstream of anti-apoptotic BCL-2-like proteins while *Ec*OmpC had no effect (Fig 1B). AlphaFold predictions show that both *Ctr*OmpA and *Sn*OmpA have the typical β-barrel structure of porins (S1B Fig). *Sn*OmpA is lacking certain stretches of *Ctr*OmpA, and these parts are in the loop regions outside the β-barrel structure (further discussed below). The identified anti-apoptotic activity of *Sn*OmpA, together with apoptosis inhibition by *Simkania negevensis* infection, is consistent with the hypothesis that OmpA is a relevant anti-apoptotic factor during infection with Chlamydia.

### Interaction of ectopically expressed OmpA and BAK on mitochondria

**Fig 2:**
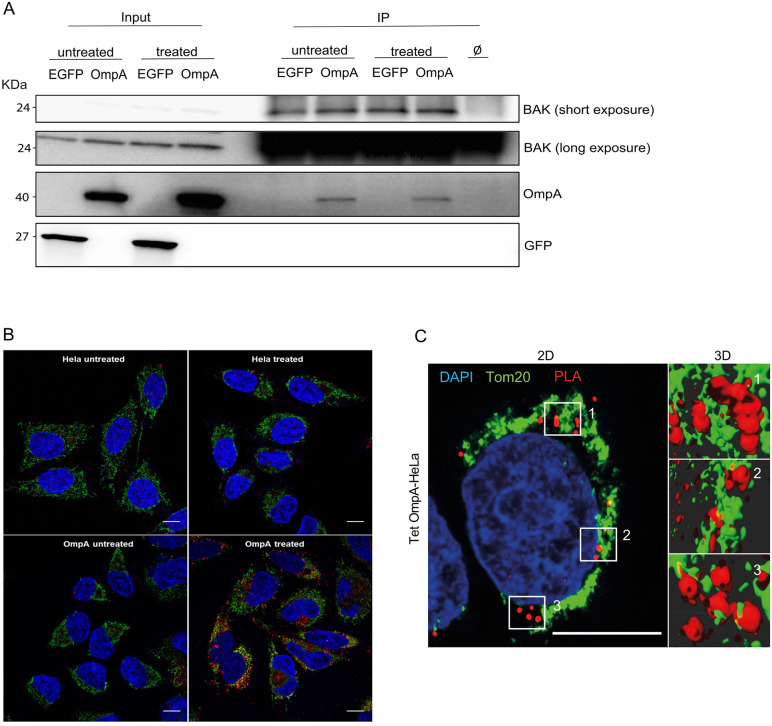
Interaction between OmpA and BAK on mitochondria. **A**, HeLa EGFP expressing cells and HeLa OmpA expressing cells were treated with ABT-737 (1 µM) and S63845 (500 nM) for 3h in the presence of the caspase-inhibitor QVD-OPh (10 μM; to block cell death downstream of mitochondria). BAK was immunoprecipitated with an antibody recognizing active and inactive BAK. Proteins were run on SDS-PAGE, and BAK and OmpA were detected by immunoblotting. Aliquots of the input and IP-reactions were loaded separately. Right lane shows beads with no antibody. Data are representative of three independent experiments. **B**, control HeLa cells (upper panel) and Tet-OmpA HeLa cells (carrying a tetracycline/AHT-inducible OmpA [12], bottom panel) were seeded on cover slips. 48h post-stimulation with AHT, cells were treated with ABT-737 (1 μM) and S63845 (500 nM) for 4h in the presence of the caspase-inhibitor QVD-OPh (10 μM). Cells were fixed, permeabilized and processed for PLA with antibodies against BAK (aa23-38; active BAK) and OmpA (red). Mitochondria were labeled using antibodies directed against TOM22 (green), and DNA was stained with Hoechst dye (blue). Confocal microscopy was performed and the overlay shows the co-localization of the PLA-signal with the mitochondrial protein TOM22. Images were acquired under identical conditions and exposure times. Data are representative of three independent experiments. Scaling bar, 10 μm. A control experiment with BAK-deficient cells is shown in S2 Fig. **C**, same set-up as in B. HeLa cells carrying a tetracycline-inducible OmpA were incubated with 100 nM AHT for 48 h to induce OmpA expression. Cells were fixed, permeabilized and processed for PLA using a different antibody specific for BAK (Ab-1(TC-100; active BAK)) and OmpA (red). Mitochondria were labeled using antibodies directed against TOM20 (green), and DNA was stained with Hoechst dye (blue). Cells were imaged by confocal microscopy. Stacks were then processed with the deconvolution software AutoQuantX and 3D reconstruction analysis was performed using the Imaris software. Images were acquired under identical conditions and exposure times. Left panel represents a single Z-slice, right panels indicate 3D reconstructions of the indicated areas using multiple Z-slices. Data are representative of three independent experiments. Scaling bar, 10 μm.

We have previously reported that OmpA, when expressed in human cells in the absence of *Ctr* infection, inserts into mitochondrial membranes and can interact with BAK [12]. During activation, BAK undergoes a conformational change exposing its N-terminus [18]. At the time, the available anti-BAK antibodies only recognized active BAK, and we could show the interaction between OmpA and BAK only during the induction of apoptosis. More recently, an antibody against total BAK, including BAK in its inactive state, has become available [19]. Using this antibody for immunoprecipitation, we found that OmpA bound to BAK also in its inactive state, in the absence of an apoptotic stimulus (Fig 2A). This is similar to the constitutive interaction between BAK and VDAC2 and again supports the view that OmpA can replace VDAC2 in its BAK-inhibitory function. To test the BAK-OmpA interaction further we used proximity ligation assay (PLA). This assay utilizes antibodies to identify close interactions of the antibody targets inside cells (within a range of 30–40 nm) [20]. We detected a clear signal of the proximity of BAK and ectopically expressed OmpA, while control PLA staining on *Ctr* infected BAK knockouts showed no signal (Figs 2B and S2) on the mitochondria of cells, as shown by co-localization with the mitochondrial outer membrane protein TOM20 (Fig 2C). These results confirm the finding that OmpA, when expressed in human cells, localizes to mitochondria and directly interacts with BAK in resting and activated states.

### OmpA translocates to mitochondria during *Ctr* infection

To inhibit BAX/BAK, OmpA has to reach mitochondria during infection. We first tested whether the interaction of OmpA with BAK could be confirmed during infection in an unbiased proteomic screen of interaction partners. We infected HeLa cells with *Ctr* and treated them with an apoptotic stimulus (staurosporine) or not. For this screen, we used two different conformation-specific antibodies that both recognize only active BAK and should isolate BAK only when apoptosis is induced; this requirement for BAK activation therefore provides an additional specificity control for the immune-precipitation. We immuno-precipitated BAK and analyzed the isolated proteins by mass spectrometry (S3A Fig). As expected, the amount of BAK detected was much greater upon apoptosis induction than in untreated cells (S3B Fig). Although numerous proteins were found in the four analyses, the only chlamydial protein that was consistently detected in all conditions was OmpA, and its identified levels were substantially greater in the samples from apoptotic cells (S3B Fig). Western blotting of immuno-precipitates from *Ctr*-infected cells treated with BH3-mimetics confirmed the co-purification of OmpA with BAK during infection and apoptosis induction (S3C Fig).

**Fig 3:**
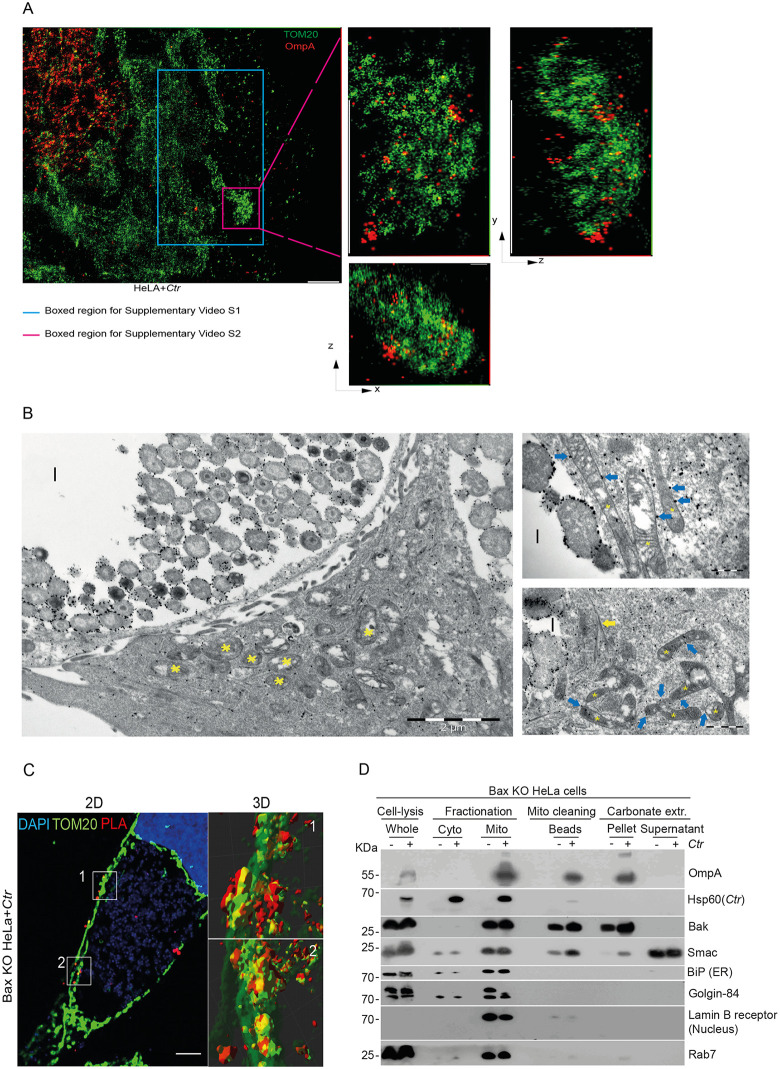
OmpA is detected at mitochondria during *Ctr* infection. **A**, 24 h after *Ctr*-infection, HeLa cells on cover slips were fixed, permeabilized and stained for two-color 3D SMLM super-resolution images of mitochondria and *Ctr* using antibodies against TOM20 and OmpA (left panel). The panels in the middle and to the right show magnified views of the pink boxed region. The x, y, and z- dimensions of the magnified region are 830 nm x 1490 nm x 780 nm. Scale bar: 1 μm. Note the localization of OmpA (red) on mitochondria TOM20 (green) during *Ctr*-infection. Insets show different perspectives of the pink boxed region. Also see S1 and S2 Movies. **B**, immuno-gold electron microscopy identifies OmpA outside chlamydial inclusions and on mitochondria. HeLa cells were infected with *Ctr* and then fixed and stained for OmpA using immuno-gold particles and were imaged using electron microscopy as described in the methods. Yellow asterisks represent mitochondria, yellow arrows indicate cytoskeletal structures and blue arrows show gold particle labelling of OmpA on mitochondrial outer membranes. I (inclusion). Note the dense labeling of the bacteria as well as the labeling of mitochondrial membranes. Scale bar references are indicated. Data represents one experiment. **C**, BAX-deficient HeLa cells were seeded on cover slips. 24 h post-infection (MOI = 5), cells were treated with ABT-737 (1 μM) and S63845 (500 nM) for 4 h in the presence of the caspase inhibitor QVD-OPh (10 μM). Cells were fixed, permeabilized and processed for PLA using antibodies against BAK (Ab-1(TC-100); active BAK) and OmpA. The red signal (PLA) indicates close proximity of BAK and OmpA. Mitochondria were stained using antibodies directed against TOM20 (green), and DNA was stained with Hoechst dye (blue). Cells were imaged by confocal microscopy. Stacks were then processed with the deconvolution software. AutoQuantX and 3D analysis was performed using the Imaris software. Left panel represents a single Z-slice, right panels indicate 3D reconstructions of the indicated areas using multiple Z-slices. Data are representative of three independent experiments. Scale bar, 5 μm. **D**, BAX-deficient HeLa cells were either *Ctr*-infected (MOI = 5) or mock-infected. 24 h post-infection, cells were fractionated, and mitochondria were purified from heavy membrane fractions using magnetic beads labelled with anti-TOM20 antibodies. Purified mitochondria were subjected to sodium carbonate extraction (pH 11.5) to separate integral from attached membrane proteins. Membranes were pelleted, and the fractions were run on SDS-PAGE. Mitochondrial membranes and membrane-integrated proteins are found in the pellet fractions. Proteins were detected using the indicated antibodies. Chlamydial HSP60, BiP (ER), golgin-84 (golgi-apparatus), lamin B (nuclear envelope) and Rab7 (endosomes) were used as organelle markers. Release of SMAC shows extraction efficiency. Data are representative of three independent experiments.

We then used single-molecule localization microscopy (SMLM) of infected BAX deficient cells to confirm the presence of OmpA outside the chlamydial inclusion and identified OmpA staining in TOM20 positive areas (i.e., mitochondria) (Figs 3A and S4). For these and further experiments where indicated, we used BAX deficient cells to remove potential confounding results due to potential interactions of OmpA with BAX. We have previously published that both BAK and BAX are regulated by OmpA and these proteins are known to interact and function with each other. To simplify the analysis of whether OmpA is directly interacting with BAK the absence of BAX removes a potential confounding factor. Immuno-gold labelling and transmission electron microscopy (TEM) further confirmed the presence of OmpA in the cytosol and on mitochondria, consistent with the interpretation of a localization of OmpA on the outer mitochondrial membrane (Fig 3B). Uninfected control cells showed very little immune gold signal supporting the specificity of the staining (S5A, S5B Fig). Of note was that additional OmpA positive signals could be seen aligning along cytoskeletal structures as indicated (Figs 3B and S5C).

We performed PLA of infected cells, again using antibodies against BAK and OmpA. As in OmpA-expressing cells, we found a clear PLA signal in close proximity of the mitochondrial protein TOM20 (Fig 3C). Next, we isolated mitochondria from *Ctr*-infected cells. Following the fractionation of cells into cytosol and heavy membrane fraction (containing mitochondria), mitochondria were purified from this fraction using anti-TOM20 immuno-purification with antibody-labelled beads. Following this purification, we obtained mitochondria (identified by the mitochondrial membrane protein BAK and the mitochondrial intermembrane space protein SMAC), with no detectable ER (BIP) and with minimal contamination from nuclear membrane (lamin B) and endosomal compartments (RAB7). OmpA very clearly co-purified with mitochondria (Fig 3D). To distinguish membrane insertion from other association with mitochondria, we extracted the mitochondria with sodium carbonate. This treatment removes any non-inserted, attached proteins before mitochondrial membranes are pelleted. OmpA was found exclusively in the membrane pellet while this disruption of mitochondria released the soluble intermembrane space protein SMAC into the supernatant as expected (Fig 3D). These results show that during *Ctr*-infection OmpA translocates to mitochondria where it inserts into mitochondrial membranes. Our previous study showing that OmpA can block mitochondrial apoptosis are thus further supported by the finding that OmpA is present at mitochondria and can interact there with BAK in both *Ctr* infected cells and cells expressing OmpA.

### OmpA resides in membrane vesicles

**Fig 4:**
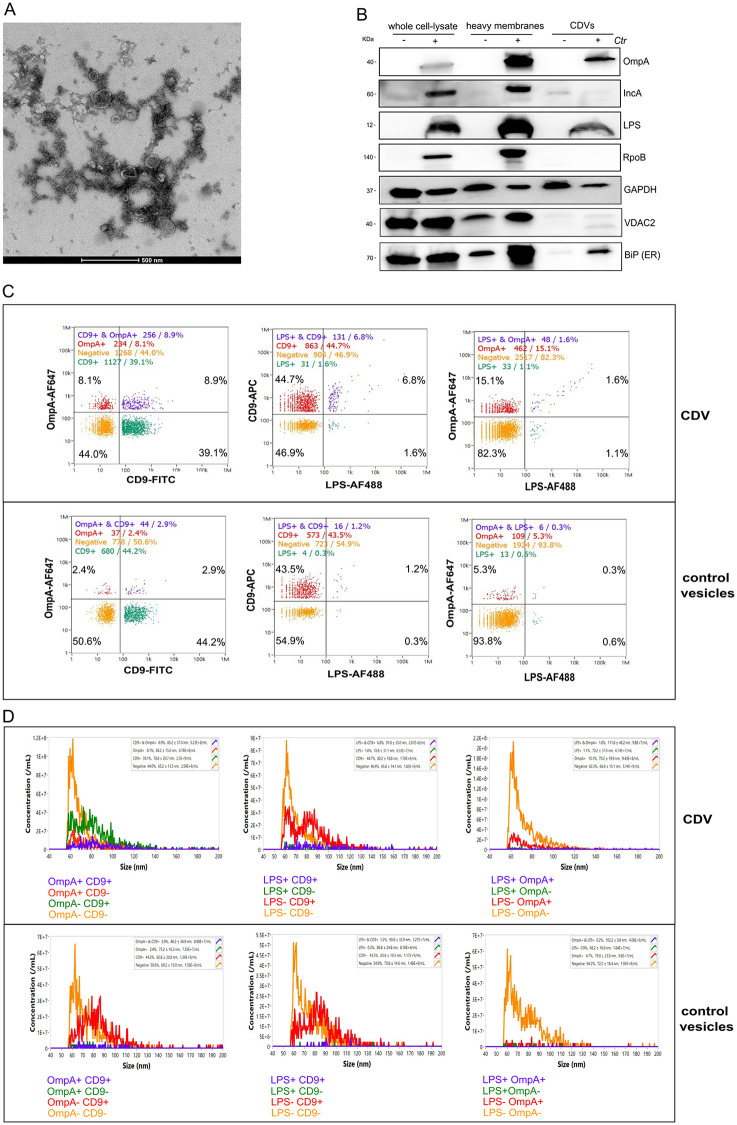
OmpA resides in membrane vesicles. **A**, electron microscopy was performed on chlamydial derived membrane vesicles, which were isolated from *Ctr*-infected HeLa cells. **B**, CDVs were isolated from *Ctr*-infected (MOI = 5, 48h) HeLa cells. Uninfected cells were subjected to the same procedure. Isolated CDV-containing fractions were subjected to Western blotting. OmpA and IncA but not the chlamydial RNA polymerase B (RpoB, as a marker for bacteria) was seen in vesicles purified from *Ctr*-infected HeLa cells. VDAC was used to detect mitochondrial contamination. BiP was used to detect ER. GAPDH was used as a loading control. Data are representative of three independent experiments. **C,** Nanoflow cytometry (nFCM) analysis shows CDVs and control vesicles that were stained by immunofluorescence staining for specific surface markers such as OmpA, LPS and EV-derived host cell marker CD9. **D,** Histograms from Nanoflow cytometry (nFCM) analysis showing size distribution of CDVs and control vesicles stained by immunofluorescence staining for specific surface markers such as OmpA, LPS and EV-derived host cell marker CD9.

The most likely way how OmpA could reach mitochondria seemed transport on vesicles, probably originating from the chlamydia themselves. We will refer to these as Chlamydia Derived Vesicles (CDVs). Transport via vesicles could also explain the OmpA immune gold signal colocalizing with cytoskeleton structures seen in the EM imaging (Figs 3B and S5C). To test this, we used OMV purification protocols established with bacteria such as *E.coli* to isolate vesicles from lysates of *Ctr*-infected cells. TEM of CDV isolations showed some vesicles like membranes (Fig 4A). Western blotting of CDVs from *Ctr*-infected cells showed OmpA and LPS but neither bacterial RNA polymerase B (RpoB) or Inclusion membrane Protein A (IncA) were detectable by western blot, indicating that no bacterial cells and little inclusion membrane had been co-purified. Likewise, no mitochondrial VDAC was detectable (Fig 4B). Both the control and *Ctr* preparations also contained some GAPDH and BIP indicating the presence of host cell membranes such as ER which is to be expected based on the cell disruption required for CDV isolation.

We performed Nano Flowcytometry (nFCM) on the samples after staining with antibodies against both the host EV marker CD9 and the chlamydial outer membrane markers OmpA and/or LPS (Fig 4C, 4D). The purification yielded vesicular structures of about 60–120 nm which is within the typical size range of OMVs and EVs (Figs 4D and S6A–S6C). Although control isolations (the same procedure performed on lysates from uninfected cells) also yielded particles (Fig 4C, 4D), no vesicular structures could be detected by TEM on these control isolations (10.6084/m9.figshare.31157629). nFCM of the samples showed that in vesicles isolated from C*tr-*infected cells, there was a clear increase in vesicles positive for OmpA and or LPS above the background (isotype staining controls or uninfected control isolations) (Figs 4C and S6A–S6C). Curiously, there was a mixture of both OmpA/CD9 (CD9 is a host membrane tetraspanin protein) double positive and also LPS/CD9 double positive as well as OmpA/LPS double positive and vesicles single positive for OmpA or LPS. This suggests that there may be fusion events between chlamydial CDVs and host membranes. It is however clear that the CDV preparations do contain what are likely outer membrane derived vesicles as well as host derived vesicles and other membranes. The size distribution of these vesicles is within the commonly found range for OMVs and EVs (Figs 4D and S6A–S6C).

Additionally, these samples were submitted to proteomics analysis for more sensitive characterization (S1 Table). Numerous Chlamydial proteins could be detected in the CDV fractions with OmpA being the second most abundant detectable protein (S1 Table). Of the other top hits in terms of quantity, the majority are either outer membrane proteins, likely outer membrane proteins or substrates of the type III secretion system which include effector proteins such as the protease CPAF and also various predicted inclusion proteins. This may reflect the presence of some inclusion membranes in the preparations, but could also be explained by CDVs containing periplasmic proteins and substrates of the type III secretion system within their lumens/membranes. Together these results are highly suggestive of the samples containing vesicles derived from *Ctr* outer membranes. Without further characterization we will however continue to refer to these isolated structures as CDVs.

### CDVs traffic to mitochondria after exogenous addition

**Fig 5:**
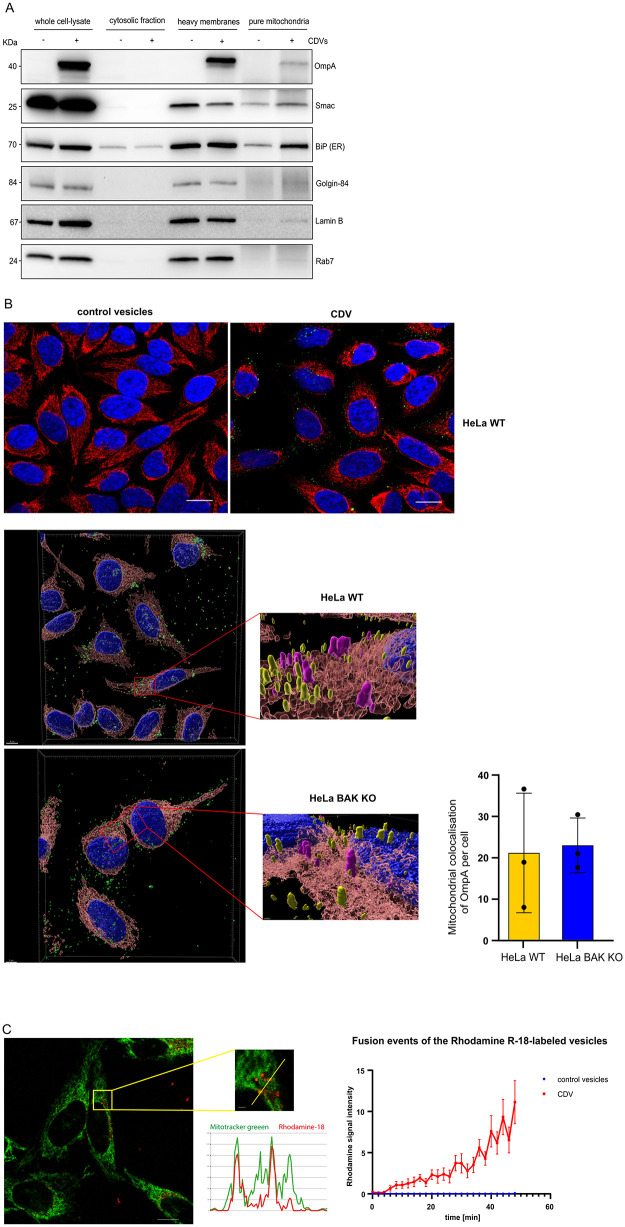
CDVs colocalize with mitochondria. **A,** HeLa cells were incubated with CDVs or control vesicles for 6h. Cells were fractionated, and mitochondria were purified from heavy membrane fractions using magnetic beads labelled with anti-TOM20 antibodies. Purified mitochondria and the fractions were run on SDS-PAGE. Proteins were detected using the indicated antibodies. Chlamydial OmpA, BiP (ER), golgin-84 (Golgi-apparatus), lamin B (nuclear envelope) and RAB7 (endosomes) were used as organelle markers. SMAC shows intact mitochondria. TOM20 (mitochondria). **B,** HeLa cells were seeded on cover slips. Cells were incubated with chlamydia derived vesicles or fractions isolated from uninfected control cells for 4h. Cells were fixed and permeabilized. Mitochondria were labeled using antibodies directed against TOM20 (red, outer mitochondrial membrane), OmpA (green), and DNA was stained with DAPI (blue). Confocal microscopy was performed with Zeiss LSM 880 and image analysis was performed using the Imaris software. 3D reconstructions of the indicated area using Z-slices were performed with Imaris software and the overlay shows the co-localization of the chlamydial vesicles with the mitochondrial protein TOM20. OmpA signal colored in pink indicates CDVs that are in contact or overlap with mitochondria. OmpA signal in yellow indicates no direct contact to mitochondria. Images were acquired under identical conditions and exposure times. Data are representative of three independent experiments*.* Scale bar, 8 μm. **C,** CDVs were labelled with rhodamine B chloride (R-18) (red) and were added to uninfected HeLa cells. Live cell imaging was performed for 50min on the confocal microscope Zeiss LSM 880. Mitotracker green was used to stain mitochondria. Graph displays fusion events of the R-18-labelled CDVs with host cell membrane over time. Scale bar, 10 μm. Data are means/SEM from three independent experiments. Control CDVs refers to fractions isolated from uninfected HeLa cells.

OMVs from other bacteria can enter mammalian cells with clear functional outcomes. We added CDVs to cells and isolated mitochondria by immune precipitation with anti-TOM20 and magnetic beads (Fig 5A). These mitochondrial fractions from CDV treated HeLa cells also show enriched levels of OmpA as well as the mitochondrial protein (SMAC). They also still contain identifiable amounts of BIP (ER). This is a common problem with mitochondrial isolations and is likely a reflection of continued binding of Mitochondrial associated membranes (MAMs) from the ER. Only minimal amounts of golgin-84 (golgi apparatus), lamin B (Nuclear) or RAB7 (endosomal) are present. These data together are highly supportive of CDV treatment leading to targeting of OmpA to mitochondrial membranes.

To further confirm this, we used Airyscan confocal microscopy on CDV treated cells. When we added CDVs to uninfected HeLa cells, an OmpA signal was detectable by Airyscan confocal microscopy not only surrounding the cells, but also on or very close to mitochondria (Fig 5B), suggesting that OmpA was transported on the CDVs into the cell and interacted with mitochondria. Given the interaction of OmpA with BAK, we also tested BAK-deficient cells to test if BAK is required to recruit OmpA, but no difference could be seen in the number of OmpA structures associated with mitochondria (Fig 5B). Vesicles would require fusion to insert OmpA into mitochondrial membranes. We tested whether CDVs can fuse with host membranes using CDVs labelled with rhodamine-R18 dye. At high concentrations, rhodamine-R18 is quenched in the vesicular membrane but dilution of the dye – which occurs upon fusion with other unlabeled membranes – de-quenches it, causing red fluorescence [21]. We added labelled CDVs to uninfected HeLa cells and observed a substantial number of fusion events over 50 minutes of recording by microscopy which can be seen as red puncta (Fig 5C). Staining of mitochondria with MitoTracker Green was consistent with the view that CDVs fused with mitochondria, at least in some of the fusion events, showing that vesicles derived from *Ctr* can traffic through the cell and fuse with mitochondria (Fig 5C). Of note, using control vesicles (containing only membranes from HeLa cells), which were also labelled like chlamydial membrane preparations with rhodamine-R18, showed no fusion events, suggesting that there is a fraction in the membranes isolated from chlamydia (probably CDVs) that can fuse with membranes inside HeLa cells. The punctate nature of the staining may be reflective of the fusion process. The number of vesicles reaching with and fusing to mitochondria at any given time is probably relatively low, as seen in the confocal microscopy, and the large mitochondrial network relative to the small CDVs likely leads to strong dilution and loss of rhodamine signal in the mitochondrial network itself, but vesicles in the act of fusion may well be fluorescent. This is however technically difficult to confirm.

### CDVs deliver OmpA to BAK and inhibit apoptosis

**Fig 6:**
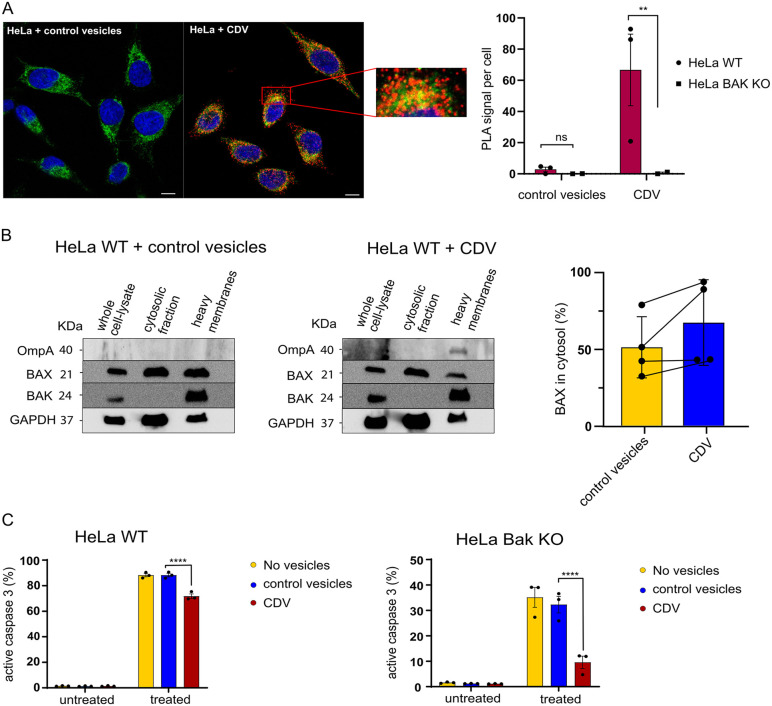
CDVs deliver OmpA to BAK and inhibit apoptosis. **A**, HeLa cells were seeded on cover slips. Cells were incubated with either chlamydial vesicles or preparations from uninfected HeLa cells (CDV control) for 1h. Cells were then treated with ABT-737 (1 μM) and S63845 (500 nM) for 3h in the presence of the caspase-inhibitor QVD-OPh (10 μM). Cells were fixed, permeabilized and processed for PLA using antibodies against active BAK (Ab-1(TC-100)) and OmpA (red) (Biozol Diagnostica, #LS-C79219). Mitochondria were labeled using antibodies directed against TOM20 (green) and DNA was stained with DAPI (blue). Confocal microscopy was performed and the overlay shows the co-localization of the PLA signal with the mitochondrial protein TOM20. Images were acquired under identical conditions and exposure times with the confocal microscope Zeiss LSM 880. BAK-deficient cells were used as a specificity control. Scale bar, 10 μm. The diagram shows the quantification from three independent experiments (columns are mean/SEM). Significance was calculated using the Kolmogorov–Smirnov test (**, p < 0.01). **B**, HeLa cells were treated with CDVs or control vesicles for 2h. Cytosolic and mitochondria-containing (heavy membrane) fractions were separated and Western blot analysis was performed. OmpA is found on the heavy membrane fraction on HeLa cells treated with CDVs. BAX retro-translocation to the cytosol is enhanced upon infection. Columns/error bars give means/SEM of four individual experiments (individual results are shown as symbols). GAPDH was used for normalization. Lines connect results of the experiments. Data are representative of four independent experiments. **C**. HeLa cells (control or BAK-deficient cells) were treated with CDVs or fractions isolated from uninfected cells (control vesicles) for 1h. Cells were then treated with ABT-737 (1 μM) and S63845 (500 nM) for 3h. Cells were fixed, permeabilized and active caspase-3 staining was performed to measure the number of apoptotic cells. Data are means/SEM of three individual experiments. Significance was tested using 2-way ANOVA (****, p < 0.0001).

We used PLA again to test for proximity of mitochondrial BAK and OmpA delivered by CDV when they were added to uninfected cells. We obtained a signal that was surprising in its clarity: in the conditions used, about 50 PLA signals could be detected per individual cell (Fig 6A). The apoptosis effector protein BAX constantly diffuses from the cytosol to mitochondria and is retro-translocated to the cytosol by BCL-X_L_ [22] and by VDAC2 [23] as an anti-apoptotic regulatory mechanism. As we reported previously, expression of OmpA in human cells also causes enhanced BAX retro-translocation, similar to the effect observed during *Ctr* infection [12]. As shown in Fig 6B, addition of CDVs from *Ctr*-infected cells also caused a redistribution of BAX from mitochondria to the cytosol, suggesting an effect of OmpA on mitochondrial BAX. We finally tested whether the addition of CDVs to uninfected cells could inhibit apoptosis. A significant reduction of BH3-mimetic-induced apoptosis by CDV addition was indeed seen (Fig 6C). Because of the effect on BAX retro-translocation, we also tested for apoptosis inhibition in BAK-deficient cells (where apoptosis relies exclusively on BAX). In these cells, the anti-apoptotic effect of CDV-addition was very strong (Fig 6C). While the effect on BAX retro-translocation was in itself not statistically significant, the trend is clear and together with the potent effect in blocking apoptosis of the CDV treatment in BAK-deficient cells points to a CDV treatment phenocopying *Ctr* infected cells. The results suggest that OmpA can travel on CDVs, insert into mitochondria through membrane fusion and inhibit apoptosis.

## Discussion

We had previously reported that OmpA, when expressed without infection, could inhibit BAK and increase the retro-translocation of BAX to the cytosol. Because these molecular effects were exactly the same as the anti-apoptotic effects identified during *Ctr* infection [12], the results suggested that OmpA may be a mediator of the anti-apoptotic effect of chlamydial infection. How OmpA may translocate to mitochondria was unclear given the significant hurdles such as its chlamydial outer membrane insertion and need to traverse the inclusion. Here we provide evidence that OmpA reaches mitochondria during infection via CDVs. This study along with our previous one support the concept that *Chlamydia* uses its relationship with mitochondria and has evolved OmpA to function as an apoptosis inhibitor, mimicking the molecular function of the mitochondrial porin VDAC2.

The porins from various bacteria can insert into mitochondria *in vitro*, as shown for Neisseria PorB [24,25], *Yersinia* YadA [26] or *E. coli* PhoE [27]. This indicates a degree of conservation between mitochondrial proteins and their bacterial ancestors. The porin from *Neisseria gonorrhoeae*, PorB, has been identified on mitochondria of infected cells, where it induced apoptosis [24], and this translocation has been suggested to occur on OMVs [16]. Surprisingly, purified PorB from *Neisseria meningitidis*, conversely, has been described to inhibit apoptosis. In that study, recombinant protein was added to human cells [28] but it is unclear how the protein would cross membranes. The ability of bacterial OMVs to enter human cells from the extracellular space is however well documented, and a number of uptake mechanisms have been proposed [29]. Mitochondrial targeting of OMVs has further been described: *E. coli* OMVs can deliver the hemolysin HlyA to mitochondria where it induced apoptosis [15]. When OMVs from *N. gonorrhoeae* were incubated with macrophages, they induced apoptosis and PorB co-localized with TOM20. Because experimental PorB expression induced apoptosis, the authors concluded that PorB on OMVs induced apoptosis in that situation. OMVs from *Acinetobacter baumannii* induced mitochondrial fragmentation, although this depended on the GTPase DRP1 and may therefore not reflect direct mitochondrial targeting of OMVs [30]. Why OMVs target mitochondria is not obvious. It is clear from this study that CDVs fuse with mitochondrial membranes but there may be fusion with other intracellular membranes as well. One possibility of how specificity could be achieved is the physical interaction between OmpA and BAK, which is inserted into the outer mitochondrial membrane; BAK recognition by OmpA may represent a targeting mechanism, however we could not show loss of association with OmpA in BAK-deficient cells after CDV incubation. It may also be an effect of different lipid composition of the intracellular, organellar membranes. The detection of hybrid CD9/OmpA double positive vesicles in our CDV preparations also may be relevant for regulating the vesicle traffic, although this remains to be shown.

Our results are at odds with a previous study investigating *Ctr* proteins at mitochondria [31]. In this study, the authors used proteomics of purified mitochondria after chlamydial infection and could detect multiple chlamydial proteins at the mitochondria, but did not report detection of OmpA. Closer investigation of the proteomics data supplied however shows that there was a strong detection of OmpA on mitochondria of infected cells, with 19 peptides representing 75% coverage of the protein and a pep score of 295 suggesting that OmpA is indeed present. OmpA was probably excluded as an artefact of the data analysis due to a low confidence peak assigned to OmpA also detected in the uninfected controls. We would therefore argue that this study actually further supports our findings that OmpA is present at mitochondria during infection.

We could previously show OmpA present on mitochondria after ectopic expression [12]. Here we show fusion events of CDVs with the mitochondrial membrane. Like with ectopic expression, OmpA is inserted into the mitochondrial membrane during *Ctr* infection, as shown by carbonate extraction (Fig 3D). When the membrane of CDVs fuses with the mitochondrial outer membrane, this can be expected directly to transfer CDV-membrane inserted OmpA into the mitochondrial membrane. A number of aspects are still unclear – how do the CDVs cross the inclusion membrane? When they fuse with the mitochondrial membrane, what will be the orientation of OmpA? To approach the question of BAK interaction, we modelled the complex of OmpA and BAK using AlphaFold [32]. Experimental mutagenesis and modelling has yielded a structural model of the interaction of BAK and VDAC2, with BAK sitting ‘on top’ of membrane-inserted VDAC2, and the BAK transmembrane domain stretching alongside VDAC2. The AlphaFold model of the complex (S7A Fig, left panel) is very similar to this in part experimentally confirmed model, with the loop identified to interact with the hydrophobic groove of BAK being in the correct place (green coloured loop on VDAC2) [33]. Intriguingly, the AlphaFold model of OmpA and BAK shows core similarities. BAK is placed similarly on top of the β-barrel, with plausible direct interaction between the proteins. Further, the BAK C-terminal transmembrane helix also extends into the membrane alongside the β-sheets (S7A Fig, right panel; AlphaFold3 cannot model membrane interactions so this structure was predicted purely for the proteins). The sequence alignment of the various OmpA proteins identified a strand-loop-strand region that was conserved between *Ctr*OmpA and *Sn*OmpA (S1A Fig, last two beta strands of alignment). In the predicted structure, this loop aligns with the transmembrane helix of BAK and may provide direct binding (S7A Fig, right panel). To test this hypothesis, we mutated the motif in *Ctr*OmpA but were unfortunately unable to achieve expression of the mutated construct in HeLa cells, even following codon optimization (not shown). The confidence scores of this heterodimer of BAK and OmpA are poor (ipTM = 0.17, pTM = 0.47). OmpA has been proposed to exist as a trimer. We therefore also modelled a trimeric OmpA complex with three BAK molecules in AlphaFold. This model shows a different orientation of the barrels in relation to BAK, with the C-terminal conserved region facing the inside of the OmpA trimer, but the alignment of the BAK transmembrane helix to the barrel of OmpA remains and the confidence score rises to within ranges that suggest these complexes may reflect the true structure (ipTM = 0.62 pTM = 0.67) (S7B Fig). With both the monomeric and trimeric models however, the N-terminal region (residues 1–35 of the mature signal peptide cleaved OmpA- coloured green) of OmpA is in position to bind into the hydrophobic groove of BAK in a similar way that VDAC2 is proposed to bind. Of note, this region is also well conserved across chlamydial species. The C-terminal half of this region is absent in *Simkania*, which also shows anti-apoptotic activity (S1 Fig), suggesting rather the N-terminal region of this loop could contain important residues for blocking apoptosis. Interestingly, this region is rich in hydrophobic residues suggesting it may fit well into the hydrophobic groove of BAK. Mutagenesis studies will be required to confirm this. Supporting this orientation of OmpA in the mitochondrial outer membrane, N terminally FLAG-tagged OmpA expressed in HeLa cells is found in mitochondrial fractions. Protease shaving of these mitochondria with proteinase K largely removed the FLAG signal in intact mitochondria, but left SMAC (an inter membrane space protein) untouched (S7C Fig). Hypotonic lysis of the outer membrane results in proteinase K mediated SMAC degradation and further sonication to break the inner membrane results in loss of the matrix localized HSP60. While this is not conclusive, it could indicate that the N-terminus of OmpA is exposed to the outside of the mitochondria, as proposed by the structural model.

The orientation of the binding in the model is noteworthy when considering how OmpA becomes inserted into the mitochondrial outer membrane. Based on this prediction, the extracellular regions of OmpA protrude into the mitochondrial intermembrane space, and the periplasmic regions will become cytosolic. If correct, this suggests that the fusion of CDVs is such that the orientation of OmpA is reversed compared to its orientation in the outer bacterial membrane. A number of ways have been proposed how OMVs form other species can fuse with human membranes, through lipid mixing or through protein interaction [29]. It is possible that the vesicles already flip over and invert when moving across the inclusion membrane or they may be delivered into the intermembrane space, as has been previously been reported for endosomes [34] where the chlamydial vesicles could fuse from the inner side of the outer membrane. Either of these pathways could provide such an inverted orientation. Various endocytic pathways are co-opted by *Chlamydia* to build its inclusion and deliver cargo into it. Multi vesicular bodies (MVBs) were shown to deliver their cargo into inclusions which is required for chlamydial growth [35,36]. This confirms that there can be vesicular traffic between the inclusion and other compartments, so it is not unlikely that these pathways could also act in the other direction. While thorough modelling or structural analysis of OmpA complexes needs to be done to confirm this model, it certainly fits with the results generated in this and our previous study showing OmpAs activity in blocking BAX/BAK activation at mitochondria.

Most bacteria are not obligate intracellular parasites and apoptosis has a minor function in the interaction of human cells and bacteria [37]. *Chlamydia* is an exception, in that it can only grow inside host cells and very likely has to inhibit apoptosis to be able to grow [6,38]. The principle of apoptosis inhibition is much clearer for viruses where anti-apoptotic activities have been known for a long time [39]. It is also easier for viruses to come up with anti-apoptotic strategies. Large DNA viruses, for instance, often carry genes whose products are similar to anti-apoptotic BCL-2-like proteins [39]; the viruses have acquired host cell genes and modified them for their purpose. Acquisition of genes from mammalian cells is not as straightforward for bacteria, and there is no evidence of horizontal transfer of mammalian genes to *Chlamydia*. We find it very interesting that *Chlamydia* appears to exploit its evolutionary relationship with mitochondria in this way. Mitochondria have acquired a number of functions in their host cell. While other apoptosis regulators are probably more important – mainly the BCL-2 family – there is a clear function in the regulation of apoptosis for VDAC2 and its interaction with BAX and BAK. *Chlamydia* appears to utilize its OmpA to inhibit apoptosis in a way that is probably very similar to the interaction of BAK with VDAC2. While OmpA has very likely essential functions in stabilizing the outer membrane through disulfide bonding and potentially as a porin, its second function in inhibiting apoptosis is certainly of great relevance and certain to contribute to its essential nature for *Chlamydia*.

## Methods

### Cell lines and cell culture conditions

HeLa 229 cervical carcinoma cells (ATCC) and 293FT cells (Invitrogen) were maintained in RPMI medium (Life Technologies, UK) supplemented with 10 % FCS (tetracycline negative; Life Technologies).

### Construct and generation of cell lines

OmpC was cloned from E. coli (NEB, #C3040H). *Simkania negevensis* (*Simk*) genomic DNA was kindly provided by Dr Matthias Horn, Vienna. OmpA from *Simk* and OmpC from *E. coli* ORFs without their respective signal peptides were amplified by PCR using the following primers: for *Simkania* (Flag-OmpA (*Simk*)_del1–18-GW-Sense; 5′-caccATGGATTACAAGGATGACGATGACAAGTTGTATAACGGCAATCCAAGT-3′ and OmpA (*Simk*)-antisense; 5′-CTAGAACTTCACTTCGCCG-3′) and for E. coli (Flag-OmpC_del1–21-GW-Sense;5′-caccATGGATTACAAGGATGA CGATGACAAGGCTGAAGTTT ACAACAAAGAC-3′ and OmpC-antisense; 5′-TTAGAACTGGTAAACCAGACC-3′). The ORFs (Gene ID: 946716 for OmpC (*E. coli*) and OmpA (*Simk*), SNE_A00410 were subsequently cloned into pENTR/SD/D-TOPO Gateway vector (Life Technologies) and were then inserted into the lentiviral vector pFCMVTO_GW_SV40_PURO_W via Gateway LR recombinase reaction (Life Technologies). Lentiviruses were used to infect HeLa cells to establish cell lines stably expressing FLAG-OmpA (*Simk*) or FLAG-OmpC. For inducible OmpA, HeLa cells were first transduced with lentivirus expressing Tet repressor, and selected cells were then infected with lentivirus carrying untagged OmpA. HeLa cells expressing either inducible or constitutive GFP were generated as above by cloning GFP into pFCMVTO_GW_SV40_PURO_W. Cells stably carrying the construct were selected with 1 μg/ml of puromycin (InvivoGen, #ant-pr-1).

### Lentivirus production and bacterial infection

For lentivirus production, 293FT cells were transfected with corresponding vectors using FuGene HD transfection (Promega, USA), following the manufacturer’s instructions. Packaging vectors were psPAX.2 and psMD2.G (Addgene Plasmids, #12260 and #12259; Didier Trono). Virus-containing supernatant was collected, filtered and incubated with HeLa cells in the presence of 1 μg/ml of polybrene (Millipore, #TR-1003-G). *Chlamydia trachomatis* serovar L2 (*Ctr*) was obtained from ATCC and propagated in HeLa cells. Bacteria were purified over a Gastrografin density gradient (Bayer Vital, Leverkusen), followed by titration on HeLa cells and stored in SPG medium (0.2 M sucrose, 8.6 mM Na2HPO4, 3.8 mM KH2PO4, 5 mM glutamic acid [pH 7.4]) at 80°C. Fresh aliquots were thawed for each experiment. Cells were infected at a multiplicity of infection (MOI) of 5 in complete culture medium without antibiotics.

### Analysis of apoptosis by flow cytometry

Apoptosis was induced by treatment with the combination of ABT-737 (Selleck Chemicals, #S1002) and the Mcl-1-inhibitor S63845 (APExBio # A8737). Cells were collected, washed and fixed with 4% paraformaldehyde (Morphisto, #11762.00250) for 10 min at room temperature, followed by staining with anti-active caspase-3 antibody (BD Pharmingen, #559565) in PBS (Life Technologies, #14190169) containing 0.5% Saponin (Roth, #4185.1) and 0.5 % bovine serum albumin (BIOMOL, #BSA-50) for 30 min at room temperature. Alexa Fluor 647-conjugated donkey anti-rabbit IgG (Dianova, #711605152) was used as secondary antibody and cells were analyzed by flow cytometry using a FACS Calibur Flow Cytometer (Becton-Dickinson, Heidelberg).

### Mitochondria isolation and determination of membrane insertion using sodium carbonate-extraction and protease shaving

A confluent 15 cm plate of uninfected HeLa cells, *Ctr* infected or cells treated with CDVs were harvested, washed with PBS and resuspended in 400µL of MB-EDTA buffer (Mannitol 210 mM, Sucrose 70 mM, EDTA 1 mM, HEPES (pH 7.5) 10 mM, Protease inhibitor (Roche) 1X). Cells were left for 20min to swell with regular vortexing. Cells were then disrupted by passing through a 27G needle using 1 mL syringe 30x or until all cells were broken open. Cell lysates were spun at 2000g for 5 min at 4 °C for 3 times and the pellets discarded. Lysates were then spun at 13,000 g for 10 min to pellet mitochondria and other heavy membranes. The supernatant was spun at 120,000 g for 1 h at 4 °C using an ultracentrifuge (Beckman Coulter Optima MAX-TL) and the supernatant taken as the cytosolic fraction. The pellet from the 13,000g spin was resuspended in 100µL of MB-EGTA (Mannitol 210 mM, Sucrose 70 mM, EGTA 1 mM, HEPES (pH 7.5) 10 mM, Protease inhibitor (Roche) 1X) and spun at 500g for 5 min and the pellet discarded. The heavy membrane fraction containing mitochondria was then spun at 10,000g for 10min, the pellet washed with 100µL of MB-EGTA buffer then spun again at 10,000g for 10min at 4 °C. The pellet was resuspended in 50µL of MB-EGTA buffer for further experiments. For pure mitochondria, the heavy membrane fractions were incubated with anti-TOM20 antibodies (SantaCruz, #sc-11415) for 2h followed by 1h incubation with pre-washed Protein-G Dynabeads (Thermo-Fischer). Where indicated, cleaned mitochondria were subjected to sodium carbonate extraction (pH 11.5) to separate integral from attached membrane proteins. Fractions were boiled in Laemmli-buffer at 95°C and run on SDS-PAGE. Mitochondrial membranes and membrane-intergrated proteins are found in the pellet fractions of carbonate extracted samples. Proteins were detected using the indicated antibodies. Chlamydial OmpA and Hsp60, BiP (ER), BAK, golgin-84 (golgi-apparatus), lamin B receptor (nuclear envelope) and RAB7 (endosomes) were used as organelle markers. Release of SSMAC shows extraction efficiency for carbonate treatment.

For protease shaving to determine orientation of OmpA, the heavy membrane fraction (roughly 30–50 µg of protein per condition) was resuspended in 150 µL of either MB-EGTA buffer or for mitoplasting, hypotonic buffer (10 mM HEPES pH 7.5.) was used. Proteinase K was added where indicated at 20µg/mL for 20min on ice. Outer membrane rupture was encouraged by regular vortexing and for disruption of the inner membrane, the heavy membranes were resuspended in hypotonic buffer and sonicated using a Biorupter set on high with 2x cycles of 30 s on, 15 s off before returning to ice for the remaining 20min. Proteinase K was inhibited at the end of the incubation by addition of PMSF solution (Sigma-Aldrich) and mitochondria were pelleted by spinning at 10,000 g for 10 min. The pellet was resuspended directly in 1x in Laemmli-buffer with PMSF and boiled at 95 °C and run on SDS-PAGE.

### Immunofluorescence and confocal microscopy

For OmpA staining, cells were seeded on coverslips and treated as indicated. After fixing for 15 min in 4% PFA, cells were permeabilized for 10 min in 0.2 % Triton X-100 (Sigma) in PBS, and incubated for an additional 30 min in 5 % BSA in PBS. Polyclonal goat anti-OmpA (Biozol Diagnostica, #LS-C79219) was used against OmpA, followed with Alexa Fluor 488-conjugated donkey anti-goat IgG (Thermo Fisher Scientific, #A-11055). To investigate mitochondrial localization of OmpA, mitochondrial protein TOM20 was co-stained with OmpA using monoclonal Mouse anti-TOM20 (SantaCruz, #sc-11415), followed by Cy5-conjugated donkey anti-mouse IgG (Dianova, #715-175-151). DNA was stained with DAPI (2 mg/ml; Sigma, #D9542) for 10 min before being mounted in Permafluor (Thermo Fisher). Images were taken using a Zeiss LSM 880 Airyscan confocal microscope at a 63x magnification (oil immersion). Z stacks were done using 45 slices and spacing between slices 0.2 µm. For visualization ZEN 3.0 (Zeiss) software was used. To investigate co-localization of OmpA with Mitochondria, 3D surfaces were reconstructed from z stacks. For that, Imaris (version 10.2, Oxford Instruments), a commercial 3D image analysis software, was used. First, for each channel, surfaces were modelled based on an adequate intensity threshold that separates the positive signal from the background. Next, OmpA derived surface objects that shared an overlapped volume greater than 0 µm^3^ with mitochondrial surfaces were counted. These OmpA objects were considered to be in direct contact with mitochondria.

### Proximity ligation assay

Cells were seeded on coverslip and infected with *Ctr* or incubated for 1 h with control Vesicles or CDVs. Cells were then treated with 1µM ABT-737 (Selleck Chemicals, #S1002) and 500nM Mcl-1-inhibitor S63845 (APExBio # A8737) for 2.5h in the presence of caspase inhibitor QVD-OPh (Gentaur (ApexBio), #GEN2269261) 10 µM. Cells were washed and fixed with 4 % PFA for 10min at room temperature. Mitochondria were first stained with polyclonal rabbit anti-TOM20 antibody (SantaCruz, #sc-11415), followed by Alexa Fluor 488-conjugated donkey anti-rabbit IgG (Dianova, #711-545-152). Duolink Proximity Ligation Assay (Sigma, #DUO92006-30RXN) was performed according to the manufacturer’s instructions. Antibodies used were monoclonal mouse anti-Bak (Millipore, #AM03) and polyclonal goat anti-OmpA (Biozol Diagnostica, #LS-C79219), followed by labelling with the corresponding secondary antibodies. After DNA staining with DAPI (Sigma, #D9542), images were taken using a Zeiss LSM 880 confocal microscope at a 63x magnification (oil immersion) and analyzed with ZEN 3.0 (Zeiss) software.

### Electron Microscopy of outer membrane vesicles

Cell lysates were fixed with 4% paraformaldehyde in 0.025 M cacodylate buffer (pH 7.4) for 1 hour at room temperature. For negative staining, 3.5 µL of the fixed sample was applied to glow-discharged 300-mesh carbon-coated copper grids (Science Service, catalog #ECF300-Cu-50) and allowed to adsorb for 60 seconds. Excess liquid was gently blotted with filter paper, and the grids were washed twice with ultrapure water. The samples were then negatively stained by applying a 10 µL drop of 2% uranyl acetate for 30 seconds, followed by blotting to remove excess stain. After air-drying at room temperature, the grids were examined using a Talos L120C transmission electron microscope (ThermoFisher Scientific) operated at 120 kV.

### Immuno-Gold electron microscopy

Cells were infected with *Ctr* (MOI = 5) for 48 h before they were fixed for 30 min at room temperature in 4% paraformaldehyde plus 0.05 % glutaraldehyde in 0.1 M phosphate buffer. Afterwards, cells were permeabilized with 0.1% Triton X-100 (Sigma) for 3 min, quenched with 0.05 % glycine for 5 min and blocked in 5% bovine serum albumin (BIOMOL, #BSA-50) for 30 min. For immune-gold labelling cells were incubated with primary goat anti-OmpA antibody (Biozol Diagnostica, #LS-C79219) in 1 % BSA at a dilution of 1:500 for 2h at room temperature. Washing steps were followed by incubation in secondary antibody coupled to 1.4 nm gold (Nanogold-Fab’ rabbit anti-goat IgG, Nanoprobes, USA, #2006) in 1 % BSA at a dilution of 1:100 for 2h at room temperature. Several washes were performed before the post fixation buffer with 4 % PFA + 2 % Glutaraldehyde in 0.1 M Sodium Cacodylate Buffer pH 7.4 was added to the cells for 15min at room temperature. Lastly, nanogold was enhanced for exactly 8 minutes using silver enhancement Kit (HQ silver, Nanoprobes, USA) in complete darkness. Cells were contrasted in 1% osmium tetroxid and 1% uranyl acetate (in 70 % ethanol) both for 30 min at RT. After dehydration, cells were embedded in epoxy resin (Durcupan, Sigma Aldrich) and ultrathin sections were cut using a Leica UC6 ultramicrotome. For imaging a Zeiss TEM 910 was used.

### Rhodamine-18 membrane fusion

CDVs were pelleted at 120,000 g for 30 min at 4°C and resuspended in labeling buffer (50 mM Na2CO3, 100 mM NaCl, pH 9.2). Rhodamine isothiocyanate B-R18 (Molecular Probes) was added to the CDVs at a concentration of 1 mg/ml for 1 h at 25°C, 300 rpm followed by ultracentrifugation at 120,000 g for 30 min at 4 °C. Rhodamine labeled-CDV were resuspended in PBS (0.2 M NaCl) and pelleted again. Labeled-CDVs were resuspended in 1 ml PBS (0.2 M NaCl) containing a protease inhibitor cocktail tablet (Complete Protease Inhibitor Tablet, Roche). R-18 labeled CDVs were added to HeLa cells. Live cell imaging was performed for 50min on the confocal microscope Zeiss LSM 880. Mitotracker green was used to stain mitochondria.

### BAK immunoprecipitation

Bak protein was immunoprecipitated as described [40]. Cells were treated with 1 µM ABT-737 and 500 nM Mcl-1-inhibitor S63845 for 3 h in the presence of caspase inhibitor QVD-OPh (Gentaur (ApexBio), #GEN2269261) (10 µM). Cells were harvested and directly lysed in buffer containing 1 % CHAPS (Carl Roth, #1479.2). Following centrifugation, protein concentration of the supernatants was quantified using Bradford assay (Bio-Rad) and equal amounts of protein lysates were loaded on the gel. Anti-BAK antibody (either clone 7D10; Walter and Eliza Hall Institute, Melbourne, Monoclonal Antibody Facility, total BAK, or clone aa23–38, active BAK) was incubated with protein G agarose beads (Roche, #11719416001) for 30min at 4°C to allow the antibody to bind to the beads. Afterwards, lysates were incubated with anti-BAK antibody and protein G agarose beads overnight at 4°C under constant agitation. Samples were collected, boiled in Laemmli buffer containing DTT for 5 min at 95°C, separated by SDS-PAGE and immunoblotted.

### Nanoflow cytometry

Nanoflow cytometry (nFCM) analysis was performed on a Nano Analyzer (NanoFCM Co., Ltd., Nottingham, UK) equipped with a 488 nm laser. The instrument was calibrated prior to measurements using 250 nm silica calibration beads (QS3003, NanoFCM Co.) at a concentration of 2.11 × 10^10^ particles/mL, which also served as a reference for determining particle concentration. Size calibration was performed using monodisperse silica beads (S16M-Exo, NanoFCM Co. Ltd.) of four different sizes (68 nm, 91 nm, 113 nm, 155 nm). Freshly filtered (0.22 µm) 1 × PBS was analyzed as a background control, and its signal was subtracted from all subsequent sample measurements. Data were acquired over a 1-minute collection time at a constant sample pressure of 1.0 kPa. For analysis, vesicle samples were diluted in 0.1 µm-filtered 1 × PBS to achieve a particle count within the optimal range of 2,500–12,000 events per minute. Particle concentration and size distribution were determined using the NF Profession v2.08 software (NanoFCM Co. Ltd.).

For immunofluorescence staining, vesicles were for specific surface markers. Samples were incubated with primary antibodies for 1h at room temperature. The following primary antibodies were used: anti-OmpA (1:1,000; LS-C79219, LifeSpan BioSciences), anti-LPS (1:100; ACI-P, Progen). Following primary staining, samples were incubated with fluorescent secondary antibodies for 1 h at room temperature: OmpA-stained samples with AF647-conjugated anti-goat IgG (705-605-147, Jackson ImmunoResearch), and LPS-stained samples with AF488-conjugated secondary antibody (115-485-062, Dianova). For CD9 detection, directly conjugated antibodies were used: APC-anti-CD9 (312108, BioLegend) or FITC-anti-CD9 (312103, BioLegend).

To ensure signal specificity, the following controls were included in each experiment: unstained vesicles, buffer-only blanks, secondary antibody-only controls (for OmpA and LPS stains), and isotype controls. For CD9 staining, the following isotype controls were used: FITC-Mouse IgG1, κ and APC-Mouse IgG1, κ. After staining, all CDV samples and controls were diluted 1:100 in PBS immediately prior to nFC acquisition.

### Preparation of *Ctr* Infected samples for proteomic analysis

For stable isotopic labeling by amino acids in cell culture (SILAC), cells were labeled with either L‑arginine (Arg0) and L‑lysine (Lys0) (‘light’ amino acids) or with 13C615N4 L‑arginine (Arg10) and 13C615N2 L‑lysine (Lys8) (‘heavy’ amino acids) (Silantes, #282986444) in DMEM supplemented with 10% dialyzed FCS and glutamine (Silantes, #282946423) for at least two weeks. Then ‘light’ labeled cells were infected with *Ctr* for 24 h and ‘heavy’ labeled cells were left uninfected. Aliquots were treated with staurosporine in the presence of caspase inhibitor (10 µM). Whole cell lysates were obtained by lysing the cells in 1% CHAPS buffer. Precleared lysates were immunoprecipitated for 120 min using either BAK(aa23–38) or BAK(Ab-1) and protein G agarose beads. Before elution, beads from *Ctr*-infected- versus uninfected-sample for the same experimental condition and same antibody were mixed. Bound fractions (eluates) were obtained by boiling the beads at 95 °C in Laemmli-buffer and were run on SDS-PAGE and processed for mass spectrometry. LC-MS/MS analysis of the in-gel digested, co-immunoprecipitated proteins was performed as described previously [41]. Data were analyzed by MaxQuant v 1.5.2.8 using a combined database, consisting of Chlamydia trachomatis serovar and human protein sequences. The false discovery rate at the peptide and protein level was 1 %. Proteomic data are available via PRIDE (https://www.ebi.ac.uk/pride/) with identifier PXD011848).

### SDS-PAGE and western blotting

Cells were lysed and boiled in Laemmli buffer (Thermo Fisher, #89900). Samples were heated at 95 °C for 5 min. Antibodies used were: BAK(NT), BAK(aa23-38), BAK(Ab-1), BAK(7D10) as above. The following antibodies were purchased from Cell Signaling unless indicated otherwise (targets): GAPDH (Millipore, #MAB374), BAX (#2772), VDAC (#4661), HSP60 (#4870), SMAC (#2954), Hsp60(*Ctr*) (Enzo Life Sciences, #ALX-804-072), OmpA (Biozol Diagnostica, #LS-C79219), FLAG (Sigma, #F1804), GFP (Roche, #11814460001), BiP (Cell signalling, #3177), golgin-84 (SantaCruz, #sc-365337), lamin B (Abcam, #ab45848), RAB7 (Abcam, #ab50533), α-tubulin (Sigma, #T9026). Peroxidase-conjugated secondary antibodies were goat anti-rabbit IgG (Sigma, #A6667), goat anti-rabbit Fc (Sigma, #AP156P), goat anti-mouse IgG (Dianova, #115035166), goat anti-mouse Fc (Sigma, #AP127P), goat anti-rat IgG (Dianova, #112035062) and mouse anti-goat (Dianova, #205035108).

### Single-Molecule Localisation Microscopy (SMLM)

For OmpA staining, cells were seeded on glass coverslips (Marienfeld, 24 mm, #1.5-H-117540) in a 6 well-plate and infected with Ctr (MOI = 5). Cells were fixed 24 h post-infection for 15 min in 4 % PFA, were permeabilized for 10 min in 0.2 % Triton X-100 (Sigma) in PBS, and incubated for an additional 30 min in 5 % BSA in PBS. Polyclonal goat anti-OmpA (Biozol Diagnostica, #LS-C79219) was used to detect OmpA, followed by AF647-conjugated donkey anti-goat (Thermo Fisher, #A21447). To investigate mitochondrial localization of OmpA, mitochondrial protein TOM20 was co-stained with OmpA using polyclonal rabbit anti-TOM20 (Santacruz, #sc11415), followed by CF680-conjugated donkey anti-rabbit (Biotium, #20418). DNA was stained with Hoechst 33342 (1 mg/ml; Sigma).

### Microscope setup and imaging for SMLM

SMLM data were acquired on a custom built widefield setup described previously. Briefly, the free output of a commercial laser box (LightHub, Omicron-Laserage Laserprodukte) equipped with Luxx 405, 488 and 638 and Cobolt 561 lasers and an additional 640 nm booster laser (iBeam Smart, Toptica) were collimated and focused onto a speckle reducer (Optotune, Dietikon, #LSR-3005-17S-VIS) before being coupled into a multi-mode fiber (Thorlabs, #M105L02S-A). The output of the fiber was magnified by an achromatic lens and focused into the sample to homogeneously illuminate an area of about 1,000 μm2. Alternatively, a single-mode fiber (Omicron, LightHUB) could be plugged into the output of the laserbox to allow TIRF imaging. The laser is guided through a laser cleanup filter (390/482/563/640 HC Quad, AHF) to remove fluorescence generated by the fiber. For ratiometric dual-color imaging of AF647 and CF680, the emitted fluorescence was collected through a high numerical aperture (NA) oil immersion objective (Leica, #HCX PL APO 160 × /1.43 NA), split by a 665LP beamsplitter (Chroma, #ET665lp), filtered by a 685/70 (Chroma, #ET685/70m) bandpass filter (transmitted light) or a 676/37 (Semrock, #FF01-676/37-25) bandpass filter (reflected light) and imaged side by side on the EMCCD camera. The color of the individual blinks was assigned by calculating the ratio of the intensities in the two channels. Astigmatism was introduced by a cylindrical lens (f = 1,000 mm; Thorlabs, #LJ1516L1-A) to determine the z position of fluorophores. The z focus was stabilized by an infrared laser that was totally internally reflected off the coverslip onto a quadrant photodiode, which was coupled into closed-loop feedback with the piezo objective positioner (Physik Instrumente). Laser control, focus stabilization and movement of filters were performed using a field-programmable gate array (Mojo, Embedded Micro). The pulse length of the 405 nm (laser intensity 27.5 W cm − 2) laser is controlled by a feedback algorithm to sustain a predefined number of localizations per frame. Coverslips containing prepared samples were placed into a custom build sample holder and 500 μl of blinking buffer (50 mM Tris/HCl pH 8, 10 mM NaCl, 10% (w/v) d-glucose, 500 μg ml–1 glucose oxidase, 40 μg ml–1 glucose catalase, 35 mM MEA) was added. To avoid a pH drift caused by accumulation of glucuronic acid in GLOX-buffers, the buffer solution was exchanged after about 2 h of imaging. Samples were imaged until close to all fluorophores were bleached and no further localizations were detected under continuous ultraviolet irradiation.

### Data analysis for SMLM

All data analysis was conducted with SMAP, a custom software written in MATLAB that is available as open source (github.com/jries/SMAP). Installation instructions are found in the README.md, and step-by-step guides on how to use the software to perform all analyses used in this manuscript are available via the Help menu.

### Fitting for SMLM

Astigmatic 3D data were fitted and analyzed as described previously (65). First, z stacks with known displacement of several (15–20) fields of view of TetraSpeck beads on a coverslip were acquired to generate a model of the experimental point spread function. This model was then used to determine the z position of the individual localizations. Free fitting parameters: x, y, z, photons per localization, background per pixel.

### Post-processing for SMLM

The x, y, and z positions were corrected for residual drift by a custom algorithm based on redundant cross-correlation. Localizations persistent over consecutive frames (detected within 35 nm from one another and with a maximum gap of one dark frame) were merged into one localization by calculating the weighted average of x, y and z positions and the sums of photons per localization and background. Localizations were filtered by the localization precision (0–20 nm) to exclude dim localizations. Additionally, poorly fitted localizations were excluded if their log-likelihood (LL) was smaller than the mean (LL)—3 × STD (LL). Superresolution images were constructed with every localization rendered as a two-dimensional elliptical Gaussian with a width proportional to the localization precision (factor 0.4). The reported mean photons per localization were calculated based on these merged and filtered localizations.

### Isolation of Chlamydial vesicles

Five confluent 15 cm plates of HeLa cells were infected with *Ctr* (MOI = 5) for 48 h. As a control the same number of uninfected HeLa cells was also used. After cultivation, cells were washed with 5 mL HSMG buffer (20 mM HEPES, 250 mM sucrose, 1.5 mM MgCl_2_, 0.5 mM EGTA) once. The cells were then lysed in 1 mL lysis buffer (HSMG buffer with 10 % percoll and protease inhibitor (Roche complete protease inhibitor cocktail)) using mechanical disruption by repeated (3 cycles of 10x) syringing through a 27G needle. Brocken cells were centrifugated at 2000 g for 5 min at 4 °C to pellet cell debris and intact cells. Lysates were further centrifuged at 16,000 g for 30 min at 4 °C to removed heavy membranes. Supernatant was filtered with a 0.45 µm followed by a 0.2 µm filter before ultracentrifugation was performed at 120,000 g for 60 min at 4 °C in a Beckman Coulter Optima XPN-100. The pellet was resuspended in 200 µL PBS.

### Proteomics of Chlamydia derived vesicles

Samples were resuspended in lysis buffer (5% SDS, 50mM triethyl ammonium bicarbonate (TEAB; Sigma, T7408), pH 7.5). Afterwards samples were sonicated using a Bioruptor device (Diagenode, Liège, Belgium). Samples were centrifuged at 13000g for 8 min and the supernatant used in the following steps. Proteins were reduced using 5 mM tris (2-carboxyethyl) phophine hydrochloride (TCEP) (Sigma; 75259) for 10 min at 95°C and alkylated using 10 mM 2-iodoacetamide (Sigma; I1149) for 20 min at room temperature in the dark. Following steps were performed using S-Trap micro filters (Protifi, Huntington, NY) following the manufacturer’s procedure. Briefly, first a final concentration of 1.2% phosphoric acid and then six volumes of binding buffer (90 % methanol; 100 mM TEAB; pH 7.1) were added. After gentle mixing, the protein solution was loaded to an S-Trap filter and spun at 2000 rpm for 0.5–1 min. The filter was washed three times using 150 μL of binding buffer. Sequencing-grade trypsin (Promega, 1:25 enzyme:protein ratio) diluted in 20µl digestion buffer (50 mM TEAB) were added into the filter and digested at 47 °C for 1 h. To elute peptides, three step-wise buffers were applied: a) 40 μL 50 mM TEAB, b) 40µl 0.2% formic acid in H2O, and c) 50% acetonitrile and 0.2% formic acid in H2O. The peptide solution were combined and dried in a SpeedVac.

Peptides were analyzed with the Evosep One system (Evosep Biosystems, Odense, Denmark) coupled to an timsTOF fleX mass spectrometer (Bruker). 500 ng of peptides were loaded onto Evotips C18 trap columns (Evosep Biosystems, Odense, Denmark) according to the manufacturer’s protocol. Peptides were separated on an EV1137 performance column (15 cm x 150 µm, 1.5 µm, Evosep) using the standard implemented 30 SPD method with a gradient length of 44 min (buffer A: 0.1 % v/v formic acid, dissolved in H_2_O; buffer B: 0.1 % v/v formic acid, dissolved in acetonitrile). Over the time of the gradient, the concentration of acetonitrile gradually increased from 0 to 90 % at a flow rate of 500 nl/min.

The timsTOF fleX mass spectrometer (Bruker, USA) was operated in the DIA-PASEF mode. DIA MS/MS spectra were collected in the range in an m/z range from 100 to 1700. Ion mobility resolution was set to 0.60–1.60 V·s/cm over a ramp time of 100 ms and an accumulation time of 100 ms. The cycle time was set at 1.8s. The collision energy was programmed as a function of ion mobility, following a straight line from 20 eV for 1/K0 of 0.6 to 59 eV for 1/K0 of 1.6. The TIMS elution voltage was linearly calibrated to obtain 1/K0 ratios using three ions from the ESI-L TuningMix (Agilent) (m/z 622, 922, 1222).

Raw data were analyzed with DIA-NN software (v. 2.0) [42]. A spectral library was predicted using a FASTA file containing the human protein sequences as of February 17th, 2025 (human-EBI-reference database, https://www.ebi.ac.uk/) and sequences of Chlamydia trachomatis as of October 6^th^, 2025 (Uniprot database; UP000000431). The false discovery rate (FDR) was set to 1%. The search was performed allowing one missed cleavage and cysteine carbamidomethylation enabled as a fixed modification. Match between runs was enabled. Quantification was performed using the label-free quantification algorithm MaxLFQ, which calculates the protein quantities as ratios from all peptide intensities.

Mass spectrometry raw data have been deposited at the ProteomeXchange Consortium (http://proteomecentral.proteomexchange.org) under the accession number PXD070895. Furthermore, all mass spectrometry proteomics datasets used and/or analysed during this study are available online at the MassIVE repository (http://massive.ucsd.edu/; dataset identifier: MSV000099942. This study did not generate new code for analysis.

## Supporting information

S1 Fig Comparisons of predicted OmpA of Chlamydia-species and Simkania negevensis. **A**, Clustal Omega sequence alignment comparisons of various chlamydia species comparing to *Simkania negevensis* and several *Rhabdochlamydia* species which show high level of similarity to *Simkania* OmpA. Red bar indicates the signal peptide, Black arrows indicate the transmembrane beta barrel forming beta strands of Chlamydia OmpA as predicted by AlphaFold seen in B. Colour is based on Clustal colouring. Protein identity and amino acid position are indicated. Note the high primary sequence identity among the different OmpA-homologous proteins. **B**, AlphaFold predictions of chalmydia OmpA (Left) and Simkania negevensis OmpA (right). Colouring is from AlphaFold based on confidence of prediction (shown with colour key).
(TIF)

S2 Fig Lack of PLA-signal in Ctr-infected BAK-deficient HeLa cells. BAK-deficient HeLa cells were seeded on cover slips and infected with *Ctr*. 24 h post-infection, cells were treated with ABT-737 (1 μM) and S63845 (500 nM) for 4 h in the presence of the caspase inhibitor QVD-OPh (10 μM). Cells were fixed, permeabilized and processed for PLA using antibodies against BAK (Ab-1(TC-100)) and OmpA. Mitochondria were labeled using antibodies directed against TOM20 (green), and DNA was stained with Hoechst dye (blue). Cells were imaged by confocal microscopy. Data are representative of three independent experiments. Scale bar, 5 μm.
(TIF)

S3 Fig BAK interacts with chlamydial OmpA. **A,** experimental design. HeLa cells were differentially labelled in SILAC media (‘light’ or ‘heavy’ amino acids, marked L or H). ‘Light’ cells were infected with Ctr (MOI = 5) for 24 h. Two aliquots of cells were additionally treated with staurosporine as indicated and lysed. Two sets of two lysates were combined as indicated. Lysates were precipitated using two different anti-BAK antibodies (both against active BAK, four IP-reactions in total), and four samples were collected. IP-products were analyzed by mass spectrometry. For analysis we focused on proteins identified in all reactions. **B,** OmpA Ibaq values correlate with BAK Ibaq values. Ibaq values of OmpA and BAK obtained by the proteomic analysis (a measure of protein abundance) are plotted against each other. Note the correlation of the values from the two experiments each where the same antibodies were used (#1 and #3, #2 and #4). **C,** Interaction between BAK and OmpA as detected by co-immunoprecipitaiton. *Ctr*-infected HeLa cells (MOI = 5) were treated with the combination of ABT-737 (1 μM) and S63845 (500 nM) for 4 h. Mitochondria were isolated, lysed in buffer containing 1% CHAPS, and lysates were subjected to immunoprecipitation using BAK (aa23–38) antibody or an IgG isotype control antibody. Interaction of BAK and OmpA was visualized by immunoblotting for BAK and OmpA proteins.
(TIF)

S4 Fig HeLa cell 3D SMLM reveals interactions between OmpA und mitochondria with nanometer-scale resolution. **A,** schematic representation of the analysis. For every OmpA localization we counted the number of TOM20 localizations that are closer than 50 nm in the lateral and 150 nm in the axial direction. OmpA localizations with less than 5 TOM20 neighbors are considered to be part of the background and OmpA localizations with more than 5 TOM20 neighbors are considered to be associated to mitochondria. **B,** OmpA is associated to mitochondria. We normalized the number of OmpA localizations at mitochondria to the number of TOM20 localizations and the OmpA localizations at the background to the area of the image to be independent of the image size. As a control, we shifted and mirrored the OmpA image and calculated the number of neighbors for this randomized negative control. We find that OmpA is associated to mitochondria, and that the number of OmpA molecules for the infected or overexpressed case is substantially larger than the background staining in the wildtype. WT (HeLa cells wt); infected (Ctr-infected HeLa cells for 24 h, (MOI = 5)); expressed (48 h OmpA expression in HeLa cells carrying a tetracycline-inducible OmpA). The data for this figure were created with the OmpA_mito_cc.m script that is included in SMAP (https://github.com/jries/SMAP).
(TIF)

S5 Fig Electron microscopy staining controls and OmpA immuno-gold labelling. **A**, TEM images of an uninfected HeLa cell. Note the minimal immuno-gold labelling present. Scale bar 2 µm **B**, zoom in of a mitochondria rich region of an uninfected HeLa cell. Again, very little OmpA immuno-gold staining is seen. Scale bar 500 nm **C,** Zoomed in TEM image of a *Ctr* infected HeLa cell stained for OmpA with immune-gold antibodies, Scale bar 500 nm. In all images yellow Asterix show mitochondria and yellow arrows indicate cytoskeletal structures.
(TIF)

S6 Fig Staining controls for Nano Flowcytometry of CDVs. Panels show measurement of the nFCM from **A**, unstained **B**, secondary only antibodies and **C**, isotype control antibodies on CDVs and control vesicles. Furthermore, there is a size distribution (in nm) and particle concentration (/mL) for each measured sample (CDV or control).
(TIFF)

S7 Fig OmpA and VDAC2 AlphaFold structures in complex with BAK. AlphaFold 3 software from DeepMind was used to predict following structures and interactions between OmpA:VDAC2 and OmpA:BAK. **A**, Shown are comparisons of VDAC2 with BAK in a 1:1 stoichiometry (left panel) and OmpA and BAK in a 1:1 stoichiometry (right panel). The highly conserved C-terminal region of OmpA is shown in yellow. The loop of VDAC2 identified to bind in the hydrophobic groove (blue) of BAK is shown in green, as is the N-terminal region of OmpA that may also bind into the hydrophobic groove of BAK. The predicted orientation of the structures are indicated. OmpA sequences used for prediction had the signal sequence removed. **B**, Shown are AlphaFold models of OmpA and BAK with a 3:3 stoichiometry. Colouring is the same as in A. The predicted orientation of the structures are indicated. AlphaFold scores (ipTM and pTM) are indicated for each model. **C**, Protease shaving of crude mitochondria from cells expressing FLAG-OmpA. Purified mitochondria were treated with proteinase K (20 µg/mL) on ice for 20 min in either isotonic buffer or hypotonic buffer to swell and break the mitochondrial outer membrane open. Sonication was also used with hypotonic buffer to break open the inner membrane. Samples were run on SDS-PAGE and probed for FLAG, SMAC (intermembrane space) and mitochondrial HSP60 (matrix).
(TIF)

S1 Table Proteomics analysis of CDVs. (XLSX)

S1 Movie See legend for Fig 3A. (AVI)

S2 Movie See legend for Fig 3A. (AVI)
