# Supplementary figures and images for: Chlamydial membrane vesicles deliver the beta barrel outer membrane protein OmpA to mitochondria to inhibit apoptosis
Source: PLoS Pathog. 2026 Feb 2;22(2):e1013247. doi: 10.1371/journal.ppat.1013247 (PMC12890168; doi:10.1371/journal.ppat.1013247)

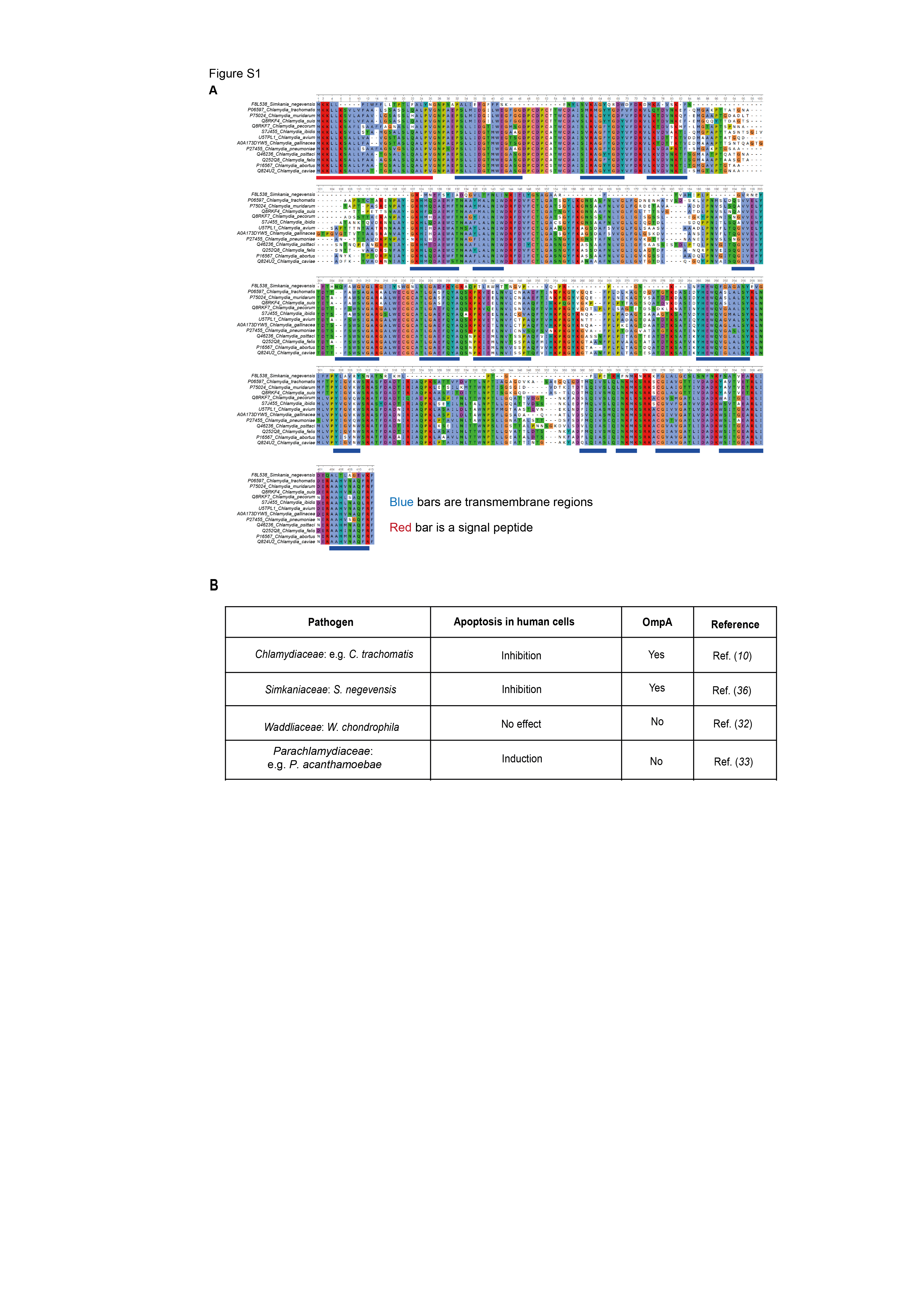

Supplement: S1 Fig — A, Clustal Omega sequence alignment comparisons of various chlamydia species comparing to Simkania negevensis and several Rhabdochlamydia species which show high level of similarity to Simkania OmpA. Red bar indicates the signal peptide, Black arrows indicate the transmembrane beta barrel forming beta strands of Chlamydia OmpA as predicted by AlphaFold seen in B. Colour is based on Clustal colouring. Protein identity and amino acid position are indicated. Note the high primary sequence identity among the different OmpA-homologous proteins. B, AlphaFold predictions of chalmydia OmpA (Left) and Simkania negevensis OmpA (right). Colouring is from AlphaFold based on confidence of prediction (shown with colour key). (TIF) [file ppat.1013247.s001.tif]

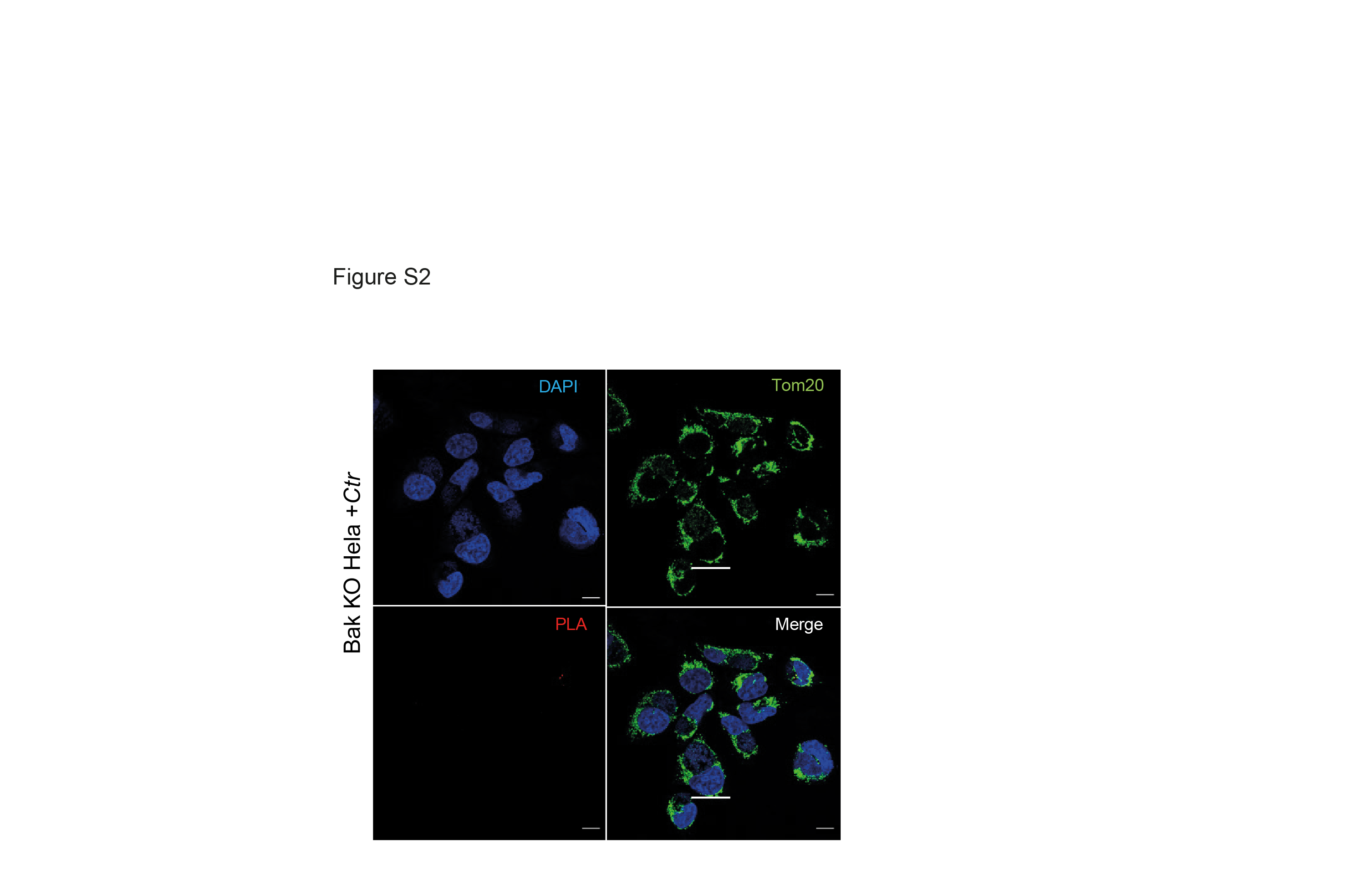

Supplement: S2 Fig — BAK-deficient HeLa cells were seeded on cover slips and infected with Ctr. 24 h post-infection, cells were treated with ABT-737 (1 μM) and S63845 (500 nM) for 4 h in the presence of the caspase inhibitor QVD-OPh (10 μM). Cells were fixed, permeabilized and processed for PLA using antibodies against BAK (Ab-1(TC-100)) and OmpA. Mitochondria were labeled using antibodies directed against TOM20 (green), and DNA was stained with Hoechst dye (blue). Cells were imaged by confocal microscopy. Data are representative of three independent experiments. Scale bar, 5 μm. (TIF) [file ppat.1013247.s002.tif]

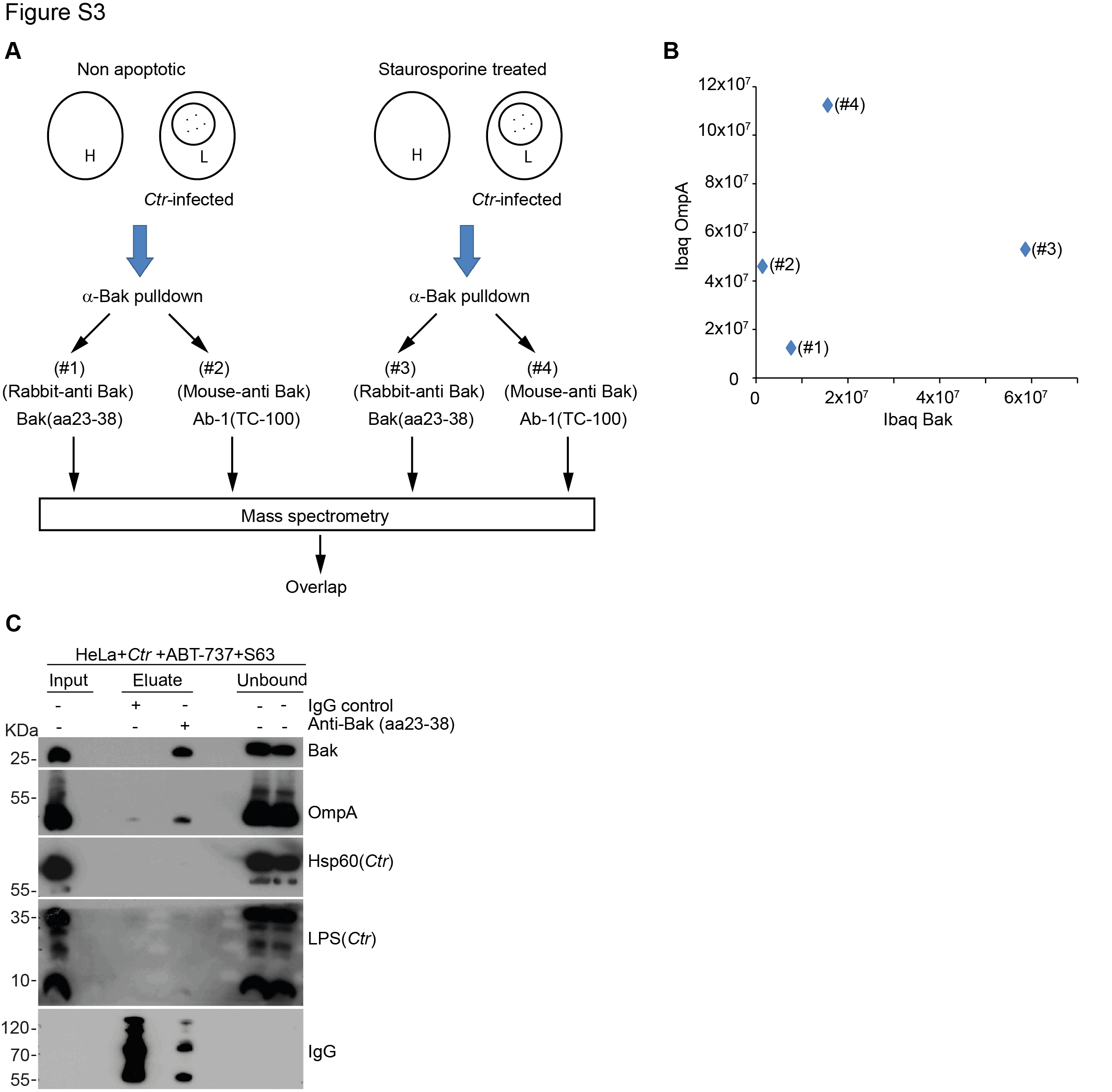

Supplement: S3 Fig — A, experimental design. HeLa cells were differentially labelled in SILAC media (‘light’ or ‘heavy’ amino acids, marked L or H). ‘Light’ cells were infected with Ctr (MOI = 5) for 24 h. Two aliquots of cells were additionally treated with staurosporine as indicated and lysed. Two sets of two lysates were combined as indicated. Lysates were precipitated using two different anti-BAK antibodies (both against active BAK, four IP-reactions in total), and four samples were collected. IP-products were analyzed by mass spectrometry. For analysis we focused on proteins identified in all reactions. B, OmpA Ibaq values correlate with BAK Ibaq values. Ibaq values of OmpA and BAK obtained by the proteomic analysis (a measure of protein abundance) are plotted against each other. Note the correlation of the values from the two experiments each where the same antibodies were used (#1 and #3, #2 and #4). C, Interaction between BAK and OmpA as detected by co-immunoprecipitaiton. Ctr-infected HeLa cells (MOI = 5) were treated with the combination of ABT-737 (1 μM) and S63845 (500 nM) for 4 h. Mitochondria were isolated, lysed in buffer containing 1% CHAPS, and lysates were subjected to immunoprecipitation using BAK (aa23–38) antibody or an IgG isotype control antibody. Interaction of BAK and OmpA was visualized by immunoblotting for BAK and OmpA proteins. (TIF) [file ppat.1013247.s003.tif]

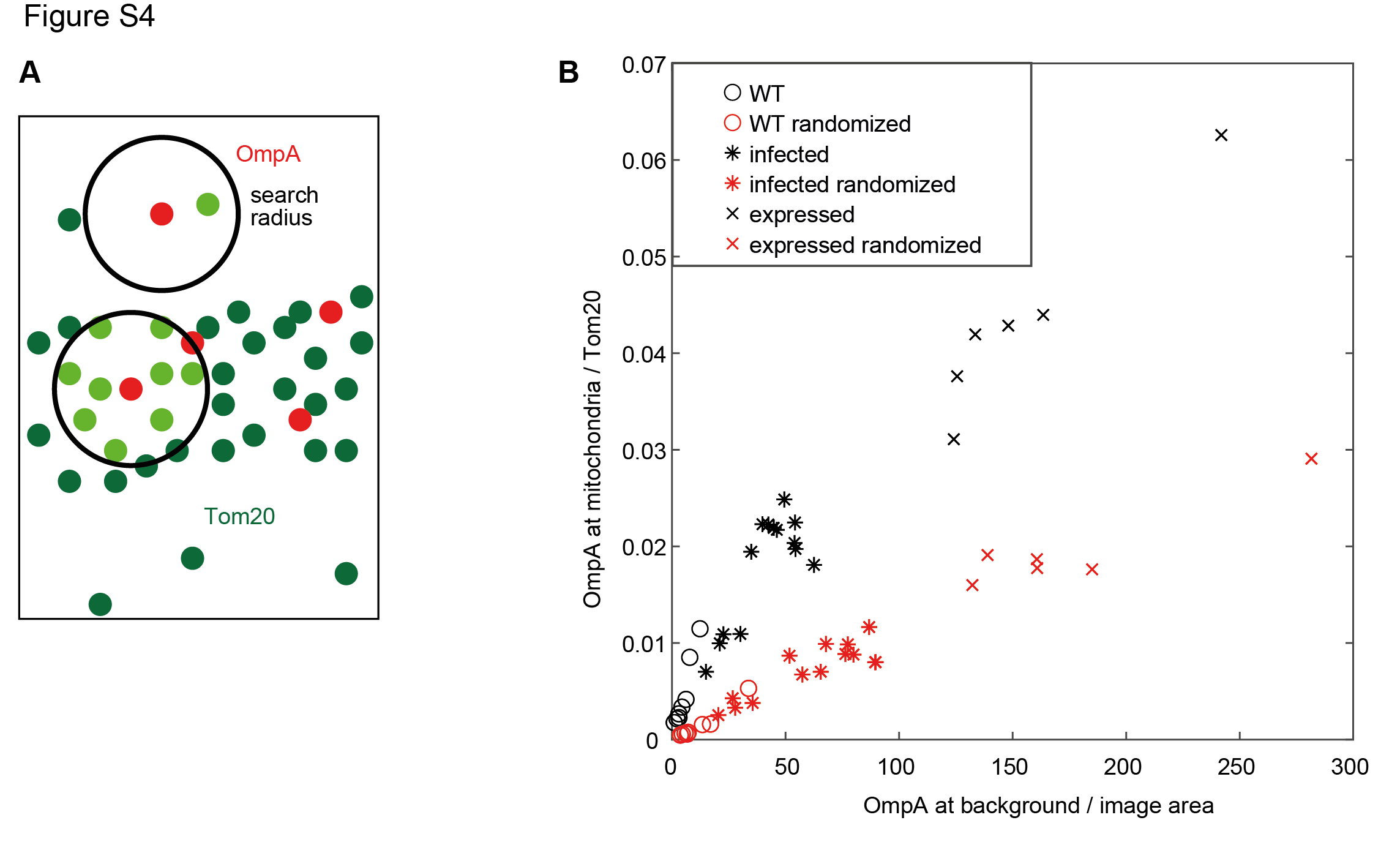

Supplement: S4 Fig — A, schematic representation of the analysis. For every OmpA localization we counted the number of TOM20 localizations that are closer than 50 nm in the lateral and 150 nm in the axial direction. OmpA localizations with less than 5 TOM20 neighbors are considered to be part of the background and OmpA localizations with more than 5 TOM20 neighbors are considered to be associated to mitochondria. B, OmpA is associated to mitochondria. We normalized the number of OmpA localizations at mitochondria to the number of TOM20 localizations and the OmpA localizations at the background to the area of the image to be independent of the image size. As a control, we shifted and mirrored the OmpA image and calculated the number of neighbors for this randomized negative control. We find that OmpA is associated to mitochondria, and that the number of OmpA molecules for the infected or overexpressed case is substantially larger than the background staining in the wildtype. WT (HeLa cells wt); infected (Ctr-infected HeLa cells for 24 h, (MOI = 5)); expressed (48 h OmpA expression in HeLa cells carrying a tetracycline-inducible OmpA). The data for this figure were created with the OmpA_mito_cc.m script that is included in SMAP (https://github.com/jries/SMAP). (TIF) [file ppat.1013247.s004.tif]

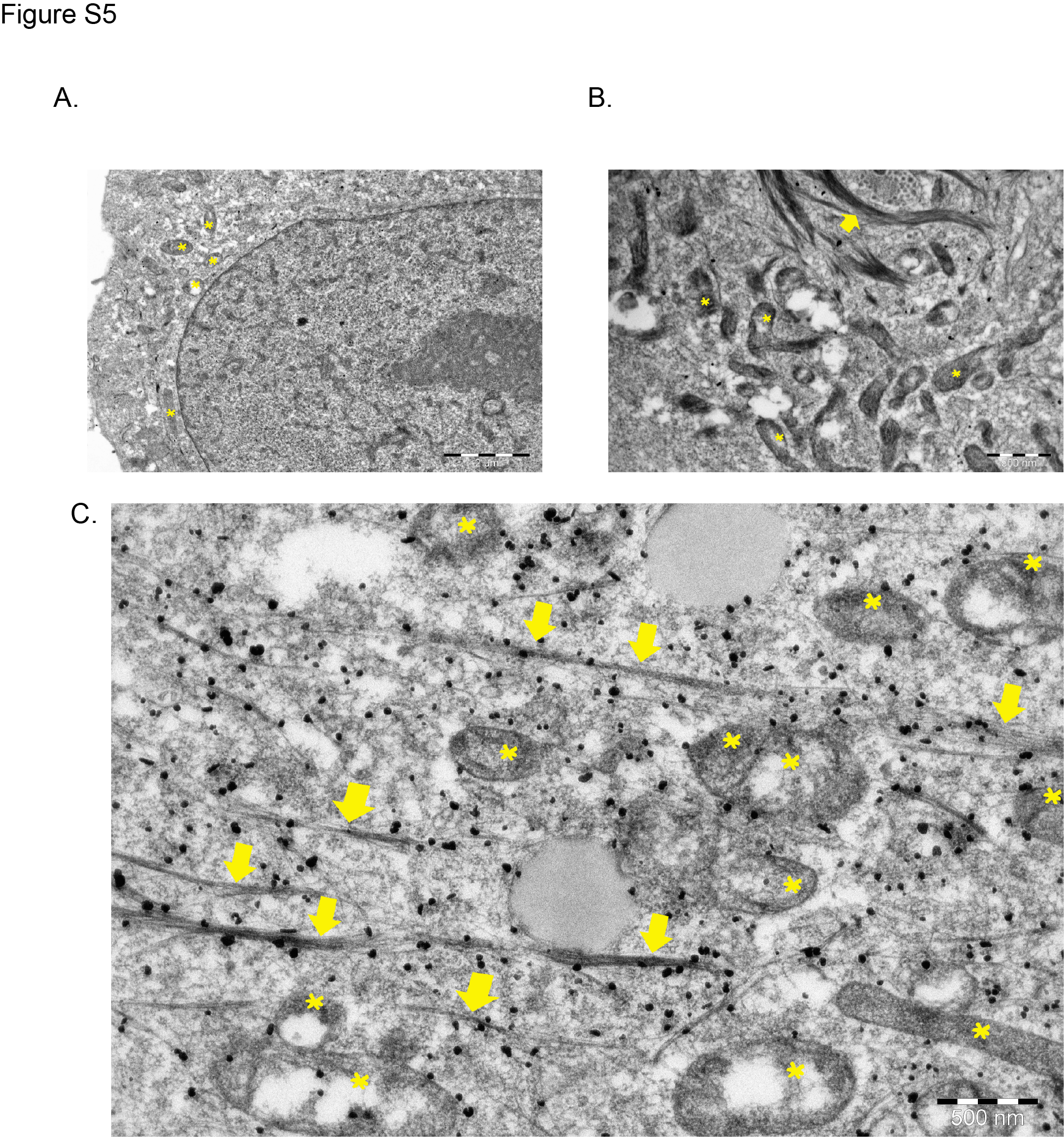

Supplement: S5 Fig — A, TEM images of an uninfected HeLa cell. Note the minimal immuno-gold labelling present. Scale bar 2 µm B, zoom in of a mitochondria rich region of an uninfected HeLa cell. Again, very little OmpA immuno-gold staining is seen. Scale bar 500 nm C, Zoomed in TEM image of a Ctr infected HeLa cell stained for OmpA with immune-gold antibodies, Scale bar 500 nm. In all images yellow Asterix show mitochondria and yellow arrows indicate cytoskeletal structures. (TIF) [file ppat.1013247.s005.tif]

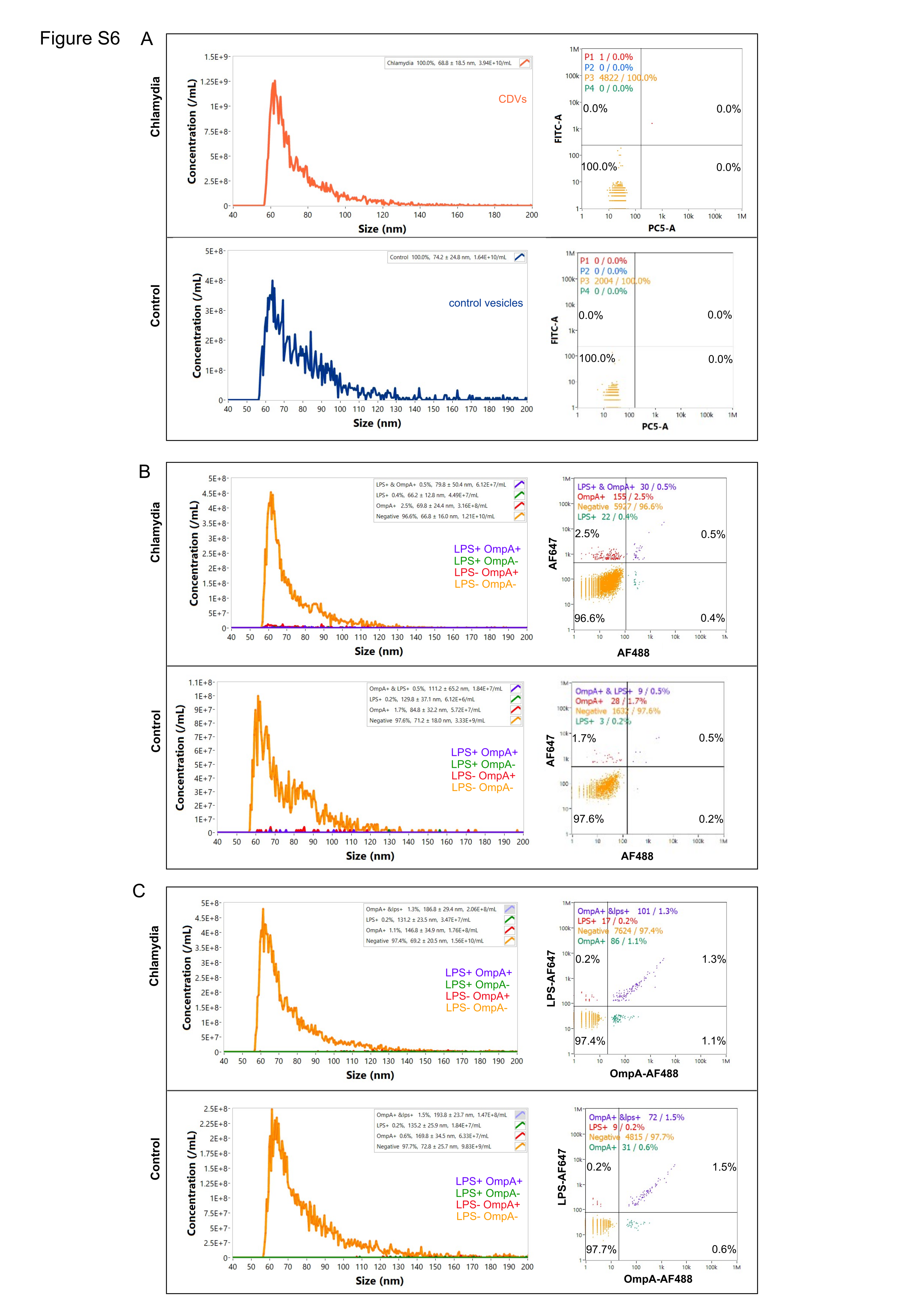

Supplement: S6 Fig — Panels show measurement of the nFCM from A, unstained B, secondary only antibodies and C, isotype control antibodies on CDVs and control vesicles. Furthermore, there is a size distribution (in nm) and particle concentration (/mL) for each measured sample (CDV or control). (TIFF) [file ppat.1013247.s006.tiff]

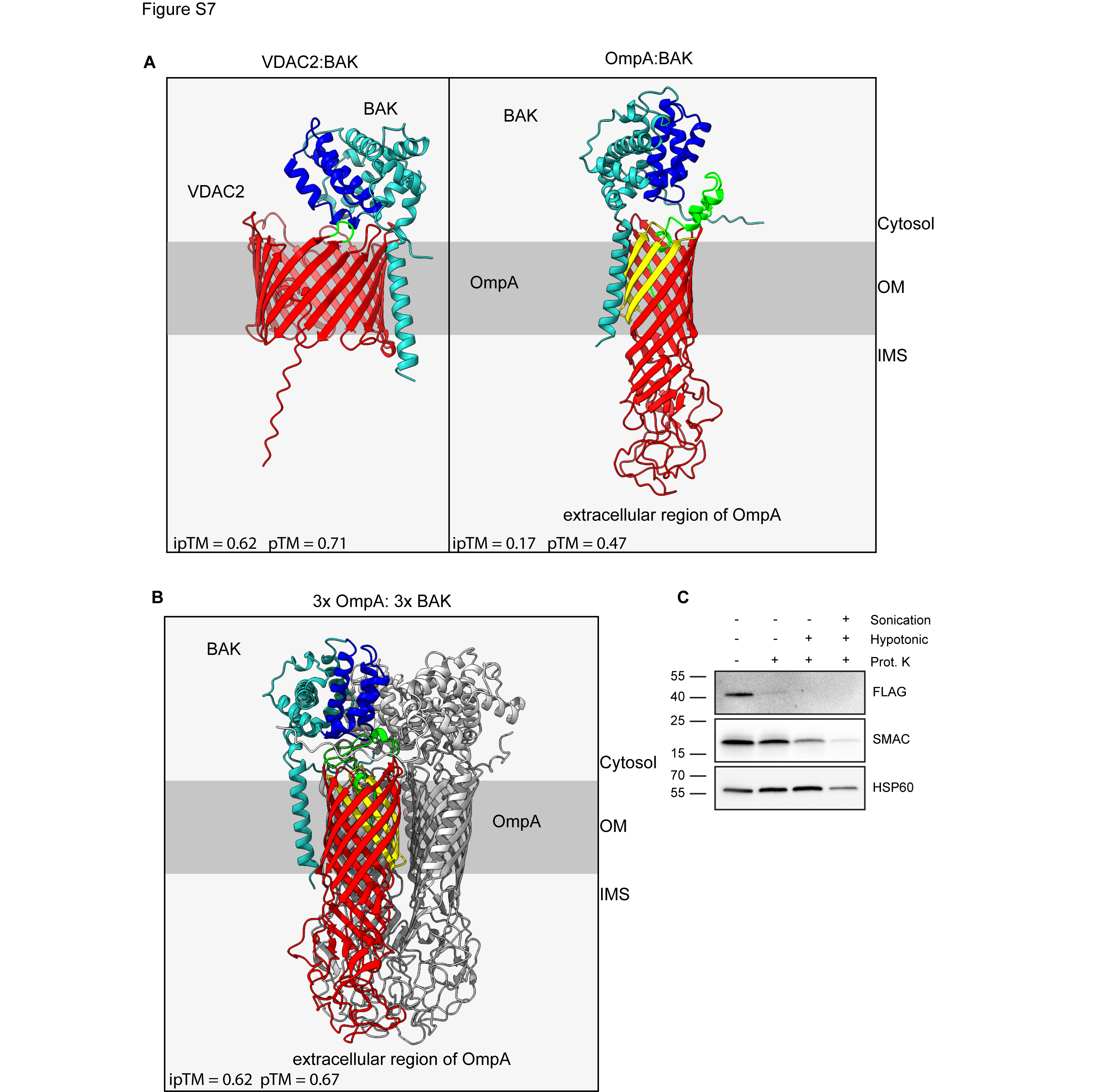

Supplement: S7 Fig — AlphaFold 3 software from DeepMind was used to predict following structures and interactions between OmpA:VDAC2 and OmpA:BAK. A, Shown are comparisons of VDAC2 with BAK in a 1:1 stoichiometry (left panel) and OmpA and BAK in a 1:1 stoichiometry (right panel). The highly conserved C-terminal region of OmpA is shown in yellow. The loop of VDAC2 identified to bind in the hydrophobic groove (blue) of BAK is shown in green, as is the N-terminal region of OmpA that may also bind into the hydrophobic groove of BAK. The predicted orientation of the structures are indicated. OmpA sequences used for prediction had the signal sequence removed. B, Shown are AlphaFold models of OmpA and BAK with a 3:3 stoichiometry. Colouring is the same as in A. The predicted orientation of the structures are indicated. AlphaFold scores (ipTM and pTM) are indicated for each model. C, Protease shaving of crude mitochondria from cells expressing FLAG-OmpA. Purified mitochondria were treated with proteinase K (20 µg/mL) on ice for 20 min in either isotonic buffer or hypotonic buffer to swell and break the mitochondrial outer membrane open. Sonication was also used with hypotonic buffer to break open the inner membrane. Samples were run on SDS-PAGE and probed for FLAG, SMAC (intermembrane space) and mitochondrial HSP60 (matrix). (TIF) [file ppat.1013247.s007.tif]
